# Folium *Sennae* protects against hydroxyl radical-induced DNA damage via antioxidant mechanism: an *in vitro* study

**DOI:** 10.1186/1999-3110-55-16

**Published:** 2014-02-02

**Authors:** Jian Lin, Xican Li, Lu Han, Fei Li, Wenbiao Lu, Ye Bai, Dongfeng Chen

**Affiliations:** 1 School of Chinese Herbal Medicine, Guangzhou University of Chinese Medicine, Waihuan East Road No.232, Guangzhou Higher Education Mega Center, 510006 Guangzhou, PR China; 2 School of Basic Medical Science, Guangzhou University of Chinese Medicine, Guangzhou, 510006 PR China

**Keywords:** Folium *Sennae*, DNA oxidative damage, Antioxidant mechanism, Hydroxyl-induced, Anthraquinones, ROS scavenging

## Abstract

**Background:**

In the study, Folium *Sennae* (FS) was firstly extracted by various solvents to obtain five FS extracts. Then, five FS extracts were evaluated for the protective effects against •OH-induced DNA damage, antioxidant abilities *in vitro,* and chemical contents using various methods. On this basis, the correlation graphs between the pharmacological effects and chemical contents were plotted to obtain the correlation coefficients (R values). Finally, in order to obtain biological evidence, ethyl acetate extract of FS (EAFS) was investigated for the protective effect against •OH-induced MSCs (mesenchymal stem cells) damage using MTT (3-(4,5-dimethylthiazol-2-yl)-2,5-diphenyl) assay.

**Results:**

The pharmacological assays indicated that five FS extracts could effectively protect against •OH-induced DNA damage. The correlation analysis suggested that the average R values of total phenolics, total anthraquinones, aloe-emodin, rhein, and emodin were respectively 0.843, 0.833, 0.753, 0.820, and 0.784, while those of total sugars and total saponins were respectively 0.103 and 0.0068. The mechanistic analysis revealed that five FS extracts could also scavenge •OH, •O_2_^–^, DPPH• & ABTS•^+^ radicals, and reduce Cu^2+^ to Cu^+^. MTT assay revealed that the viability of MSCs which were treated with •OH radicals has been effectively protected by EAFS (3 and 30 μg/mL).

**Conclusion:**

On this basis, it can be concluded that: (*i*) Folium *Sennae* exhibits a protective effect against •OH-induced damages to DNA and MSCs; (*ii* ) The effects may be attributed to phytophenols (especially aloe-emodin, rhein, and emodin), not sugars or saponins; (*iii*) They exert the protective action via hydrogen atom transfer (HAT) and/or sequential electron proton transfer (SEPT) mechanisms which make phenolic –OH moiety be oxidized to stable semi-quinone form; (*iv*) The stability of semi-quinone form can ultimately be responsible for the protective or antioxidant effect of phytophenols.

**Electronic supplementary material:**

The online version of this article (doi:10.1186/1999-3110-55-16) contains supplementary material, which is available to authorized users.

## Background

It is well known that the DNA oxidative damage by reactive oxygen species (ROS, especially hydroxyl radical •OH) can be repaired via enzymatic or non-enzymatic mechanisms, and that enzymatic repair has been widely explored. Non-enzymatic repair, however, remains relatively unknown until now. Zheng and colleagues pointed out that non-enzymatic repair plays a critic role in basic pharmacology & toxicology, because it is one billion times faster than the enzymatic repair of DNA oxidative damage. The fast non-enzymatic repair is usually exerted by natural phytophenols occurring in medicinal plants (especially Chinese herbal medicines) (Zheng et al. 2010). However, some new questions are also raised. For instance, (*i*) When a Chinese herbal medicine (medicinal plant) is used for fast repair of the DNA damage, is the repair action of phytophenols implicated with the other components? (*ii*) How and why do phytophenols exert the repair effect on DNA damage?

Since a Chinese herbal medicine Folium *Sennae* (FS) showed resistance to mutagenic effect caused by DNA oxidative damage (Silva et al. 2008; Demple and Halbrook 1983), we thus used FS as a reference plant to provide the answer to the questions.

## Methods

### Plant material and animals

Folium *Sennae* (the leaves of *Cassia angustifolia* Vahi, Additional file 1) was purchased from Caizhilin Pharmacy located in Guangzhou University of Chinese Medicine (Guangzhou, China, Lot No. YPA3A0001), and authenticated by Professor Shuhui Tan. A voucher specimen was deposited in our laboratory. Sprague-Dawley (SD) rats of 4 weeks of age were obtained from the animal centre of Guangzhou University of Chinese Medicine.

### Chemicals

Trolox (± − 6-hydroxyl-2,5,7,8-tetramethlyhromane-2-carboxylic acid), BHA (butylated hydroxyanisole), DPPH• (1,1-diphenyl-2-picrylhydrazyl radical), pyrogallol, neocuproine (2,9-dimethyl-1,10-phenanthroline) and Folin-Ciocalteu reagent were purchased from Sigma Aldrich Trading Co. (Shanghai, China); ABTS [2,2′-azino-bis(3-ethyl-benzothiazoline-6-sulfonic acid diammonium salt)] and D-2-deoxyribose were obtained from Amresco Co. (Solon, OH, USA); DNA sodium salt (fish sperm) was purchased from Aladdin Chemistry Co. (Shanghai, China); Aloe-emodin, rhein and emodin were purchased from National Institute for the Control of Pharmaceutical and Biological Products (Beijing, China). Methanol and water were of HPLC grade. Dulbecco’s modified Eagle’s medium (DMEM), foetal bovine serum (FBS) and 3-(4,5-dimethylthiazol-2-yl)-2,5-diphenyl (MTT) were purchased from Gibco (Grand Island, NY, USA); CD44 was purchased from Wuhan Boster Co., Ltd. (Wuhan, China). All other chemicals used were of analytical grade.

### Preparation of five extracts from Folium *Sennae*

**Figure 1:**
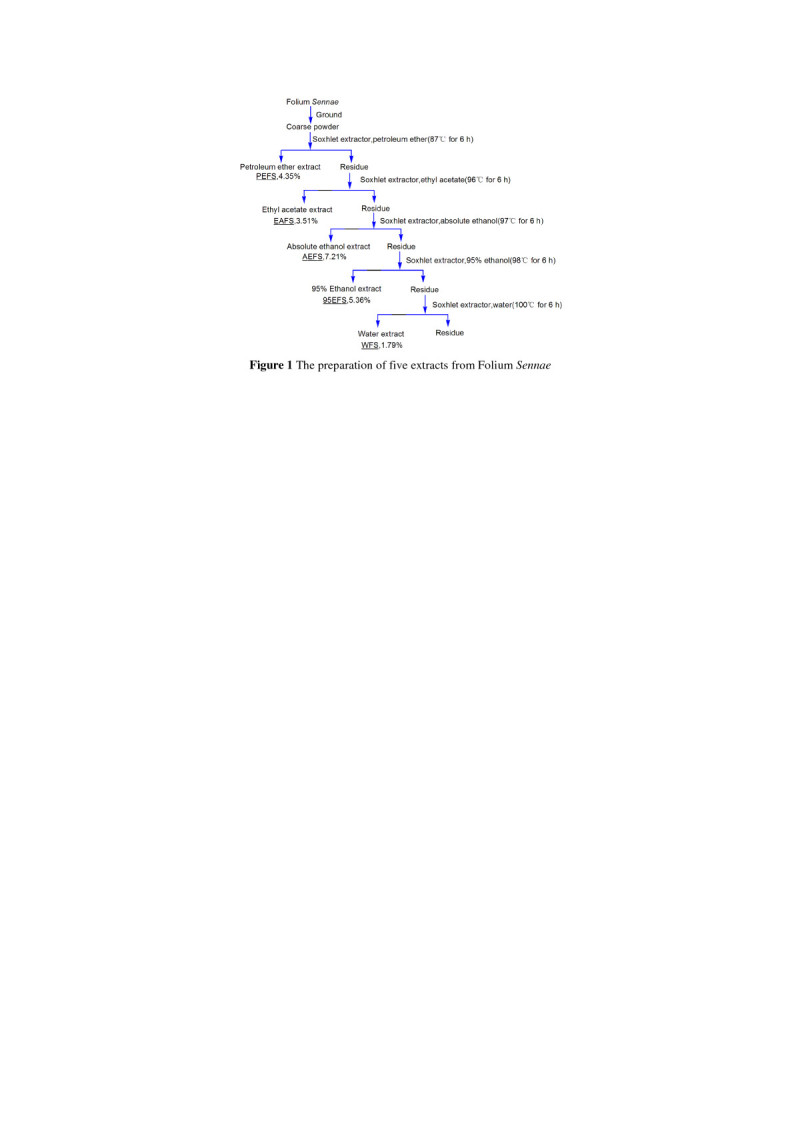
**The preparation of five extracts from Folium** ***Sennae.***

The dried Folium *Sennae* was ground into coarse powder then extracted in sequence with petroleum ether (60–90), ethyl acetate, absolute ethanol, 95% ethanol and water by Soxhlet extractor for 6 hours (Figure 1). The extracts were filtered using a Büchner funnel and Whatman No. 1 filter paper. Each filtrate was concentrated to dryness under reduced pressure at 60°C using a rotary evaporator. The dried extracts were stored at 4°C for analysis.

### Protective effect against hydroxyl-induced DNA damage

The experiment was conducted according to our method (Li et al. 2013). Briefly, sample was dissolved in methanol at 4 mg/mL. Various amounts (18–45 μL) of sample methanolic solutions were then separately taken into mini tubes. After evaporating the sample solutions in tubes to dryness, 300 μL of phosphate buffer (0.2 mol/L, pH 7.4) was added to the sample residue. Subsequently, 50 μL DNA sodium (10.0 mg/mL), 75 μL H_2_O_2_ (33.6 mmol/L), 50 μL FeCl_3_ (3.125 mmol/L) and 100 μL Na_2_EDTA (0.5 mmol/L) were added. The reaction was initiated by adding 75 μL of ascorbic acid (12 mmol/L). After incubation in a water bath at 50°C for 20 min, the reaction was terminated by adding 250 μL of trichloroacetic acid (10 g/100 mL water). The color was then developed by addition of 150 μL of TBA (2-thiobarbituric acid) (5%, in 1.25% NaOH aqueous solution) and heated in an oven at 105°C for 15 min. The mixture was cooled and absorbance was measured at 530 nm against the buffer (as blank). The percent of protection against DNA damage is expressed as follows:

$$\mathrm{Protective}\;\mathrm{effect}\;\%=\frac{A_{0}-A}{A_{0}}\times 100\%$$

Where *A*_*0*_ is the absorbance of the control without sample, and *A* is the absorbance of the reaction mixture with sample.

### Hydroxyl (•OH) radical-scavenging assay

The experiment of •OH radical-scavenging was conducted in terms of our improved method (Li 2013). In brief, the sample methanol solution (4 mg/mL, 9–36 μL) was separately added into tubes. After evaporating the sample solutions in the tubes to dryness, 400 μL of phosphate buffer (0.2 mol/L, pH 7.4) was added to the sample residue. Subsequently, 50 μL deoxyribose (50 mmol/L), 50 μL H_2_O_2_ (50 mmol/L), 50 μL FeCl_3_ (3.2 mmol/L) and 50 μL Na_2_EDTA (1 mmol/L) were added. The reaction was initiated by mixing 50 μL ascorbic acid (1.2 mmol/L) and the total volume of the reaction mixture was adjusted to 800 μL with buffer. After incubation at 50°C for 20 min, the reaction was terminated by 500 μL trichloroacetic acid (5 g/100 mL).

The color was then developed by addition of 500 μL TBA (1 g/100 mL, in 1.25% NaOH aqueous solution) and heated in an oven at 105°C for 15 min. The mixture was cooled and absorbance was measured at 530 nm against the buffer (as blank). The inhibition percentage for OH is expressed as follows:

$$\mathrm{Inhibition}\;\%=\frac{A_{0}-A}{A_{0}}\times 100\%\;$$

where, A_0_ is the A_530nm_ of mixture without sample, and A is the A_530nm_ of the mixture with sample.

### Superoxide anion (•O_2_^–^) radical-scavenging assay

Measurement of superoxide anion (•O_2_^–^) scavenging activity was based on our method (Li 2012). Briefly, the sample was dissolved in methanol at 4 mg/mL. The sample solution (*x* μL, where *x* = 0, 50, 100, 150, 200 and 250 μL) was mixed with 2950-*x* μL Tris–HCl buffer (0.05 mol/L, pH 7.4) containing Na_2_EDTA (1 mmol/L). When 50 μL pyrogallol (60 mmol/L in 1 mmol/L HCl) was added, the mixture was shaken at room temperature immediately. The absorbance at 325 nm of the mixture was measured (Unico 2100, Shanghai, China) against the Tris–HCl buffer as blank every 30 s for 5 min. The •O_2_^–^ scavenging ability was calculated as:

$$\mathrm{Inhibition}\;\%=\frac{\left(\frac{A_{325\mathrm{nm},\mathrm{control}}}{T}\right)-\left(\frac{{A_{325\mathrm{nm}}}_{,\mathrm{sample}}}{T}\right)}{\left(\frac{{A_{325\mathrm{nm}}}_{,\mathrm{control}}}{T}\right)}\times 100\%$$

Here, *ΔA*_*325nm, control*_ is the increase in A_325nm_ of the mixture without the sample and *ΔA*_*325nm, sample*_ is that with the sample; T = 5 min. The experiment temperature was 37°C.

### DPPH• radical-scavenging assay

DPPH• radical-scavenging activity was determined as described (Li et al. 2012a). Briefly, 1 mL DPPH• ethanolic solution (0.1 mmol/L) was mixed with 0.5 mL sample alcoholic solution (0.0267- 0.1333 mg/mL). The mixture was kept at room temperature for 30 min, and then measured with a spectrophotometer (Unico 2100, Shanghai, China) at 519 nm. The DPPH• inhibition percentage was calculated as:

$$\mathrm{Inhibition}\;\%=\frac{A_{0}-A}{A_{0}}\times 100\%$$

Where *A* is the absorbance with samples, while *A*_*0*_ is the absorbance without samples.

### ABTS^+^• radical-scavenging assay

The ABTS^+^• -scavenging activity was measured as described (Li et al. 2009) with some modifications. The ABTS^+^**•** was produced by mixing 0.2 mL ABTS diammonium salt (7.4 mmol/L) with 0.2 mL potassium persulfate (2.6 mmol/L). The mixture was kept in the dark at room temperature for 12 h to allow completion of radical generation, then diluted with 95% ethanol (about 1:50) so that its absorbance at 734 nm was 0.70 ± 0.02. To determine the radical-scavenging activity, 1.2 mL aliquot of diluted ABTS^+^• reagent was mixed with 0.3 mL of sample ethanolic solution (6.7- 33.3 μg/mL). After incubation for 6 min, the absorbance at 734 nm was read on a spectrophotometer (Unico 2100, Shanghai, China). The percentage inhibition was calculated as:

$$\mathrm{Inhibition}\;\%=\frac{A_{0}-A}{A_{0}}\times 100\%$$

Here, *A*_*0*_ is the absorbance of the mixture without sample, *A* is the absorbance of the mixture with sample.

### Cu^2+^-reducing power assay

The cupric ions (Cu^2+^) reducing capacity was determined by the method (Li et al. 2012b), with minor modifications. Briefly, 125 μL CuSO_4_ aqueous solution (0.01 mol/L), 125 μL neocuproine ethanolic solution (7.5 mmol/L) and (750-*x*) μL CH_3_COONH_4_ buffer solution (0.1 mol/L, pH 7.5) were brought to test tubes with different volumes of samples (1 mg/mL, *x* = 25–125 μL). Then, the total volume was adjusted to 1000 μL with the buffer and mixed vigorously. Absorbance against a buffer blank was measured at 450 nm after 30 min (Unico 2100, Shanghai, China). The relative reducing power of the sample as compared with the maximum absorbance, was calculated by the formula:

$$\mathrm{Relative}\;\mathrm{reducing}\;\mathrm{effect}\%=\frac{A-A_{\min}}{A_{\max}-A_{\min}}\times 100\%$$

where, *A*_*max*_ is the maximum absorbance at 450 nm and *A*_*min*_ is the minimum absorbance in the test. *A* is the absorbance of sample.

### Determination of total phenolics

The total phenolics contents of the five FS extracts were determined using a modified Folin-Ciocalteu colorimetric method (Li et al. 2012b). In brief, 0.1 mL sample methanolic solution (1 mg/mL) was mixed with 0.5 mL Folin-Ciocalteu reagent (0.25 mol/L). The mixture was left standing for 3 min, followed by the addition of Na_2_CO_3_ aqueous solution (1.0 mL, 15%, w/v). After standing at room temperature for 30 min, the mixture was centrifuged at 3500 r/min for 3 min. The absorbance of the supernatant was measured at 760 nm (Unico 2100, Shanghai, China). The determinations were performed in triplicate, and the calculations were based on a calibration curve obtained with quercetin. The result was expressed as quercetin equivalents in milligrams per gram of extract.

### Determination of total sugars

The content of total sugars was evaluated in terms of the phenol-sulfuric acid method (Li et al. 2012c). An aliquot of sample solution (0.2 mL, 1 mg/mL) was placed in a test tube, and the volume was adjusted to 2 mL with distilled water. Then 1 mL of 5% phenol solution and 5 mL of concentrated sulfuric acid were added. After incubation for 20 min at room temperature, the reaction mixture was measured using a spectrophotometer (Unico 2100) at 490 nm. The standard curve was prepared using different concentrations of laminarin and the results were expressed as laminarin in milligrams per gram extract.

### Determination of total saponins

The content of total saponins was measured according to the method (Li et al. 2012c). Sample methanolic solution (0.15 mL, 2 mg/mL) was taken in a test tube. After the methanol solvent was removed at 80°C, 0.1 mL vanillin-acetic acid solution (5 mg/mL) and 0.4 mL perchloric acid were added to the sample residue. The reaction mixture was incubated at 70°C for 15 min, then cooled immediately and diluted by 1.25 mL acetic acid. After 10 min, the absorbance of the diluted solution was measured at 540 nm (Unico 2100) against a blank control, which contained all reagents except for sample. Quantification was based on the standard curve for oleanolic acid (9.14–54.86 μg/mL). The results were expressed in milligrams of oleanolic acid equivalents per gram of extract.

### Determination of total anthraquinones

The total anthraquinones content was determined by the colorimetric method (Zhang et al. 2005). In brief, the sample methanol solution (0.4 mL, 4 mg/mL) was separately added into tubes. After the methanol solvent was removed at 70°C, the sample residue was dissolved with 2 mL distilled water, followed by the addition of 1 mL concentrated hydrochloric acid. The reaction mixture was incubated at boiling water for 30 min and shaken continually, then cooled and extracted by diethyl ether (10 mL/time, 3–5 times). After the extract was combined and evaporated to dryness, 6 mL NaOH aqueous solution (5%, w/v) was added and mixed vigorously. After standing at room temperature for 45 min, the reaction mixture was measured using a spectrophotometer (Unico 2100) at 520 nm. The standard curve was prepared using different concentrations of emodin and the results were expressed as emodin in milligrams per gram extract.

### HPLC analysis for aloe-emodin, rhein and emodin

Aloe-emodin, rhein and emodin in FS extracts were identified by the retention times and the peak areas were used to characterize the relative contents in the study. HPLC analysis was performed on a Syltech P510 system (Los Angeles, California, USA), equipped with a Diamonsil C18 (250 mm × 4.6 mm, 5 μm) column (Dikma Co., Beijing, China). All samples were dissolved in methanol at 10 mg/mL and filtered using 0.45 μm filters. The mobile phase consisted of methanol-0.1% phosphoric acid (85:15, v: v) and the flow rate was 0.5 mL/min, injection volume was 15 μL, detection wavelength was 254 nm.

### Protective effect against •OH-induced damage to MSCs (MTT assay)

MSCs culture was carried out according to our previous report (Chen et al 2007) with slight modifications. In brief, bone marrow was obtained from the femur and tibia of rat. The marrow samples were diluted with DMEM (LG: low glucose) containing 10% FBS. MSCs were prepared by gradient centrifugation at 900 *g* for 30 min on 1.073 g/mL Percoll. The prepared cells were detached by treatment with 0.25% trypsin and passaged into cultural flasks at 1 × 10^4^/cm^2^. MSCs at passage 3 were evaluated for cultured cell homogeneity using detection of CD44 by flow cytometry and were used for the investigation.

These MSCs were seeded at 1 × 10^4^ cells per well in 96-well plates. After adherence for 24 hr, these MSCs were then divided into normal, model, and EAFS (sample) groups. Compared with the normal group, MSCs in the model and sample groups were treated with the mixture of FeCl_2_ (100 μM) followed by H_2_O_2_ (50 μM) for 25 minutes, and then which be removed, MSCs of the normal and model groups were both cultured in serum-free DMEM (low glucose) but the sample groups were incubated with EAFS (at 3 and 30 μg/mL) which were diluted by serum-free DMEM (low glucose) for 24 hr. All groups had five independent wells. After incubation, 20 μL MTT (5 mg/mL) was added and then incubated for further 3 h. Culture medium was discarded and was replaced with 150 μL DMSO. Absorbance at 490 nm was measured by a Bio-Kinetics reader (PE-1420; Bio-Kinetics Corporation, Sioux Center, IA, USA). In the experiment, culture with serum medium was used for the control group and each sample test was repeated in five independent wells.

### Statistical analysis

All determinations were conducted in triplicate and all the results were calculated as Mean ± SD (SD). The IC_50_ values were calculated by linear regression analysis. All linear regression in this paper was analyzed by Origin 6.0 professional software (OriginLab Corporation, Northampton, MA, USA). Statistical comparisons between means were performed using one-way analysis of variance (ANOVA). Values of p < 0.05 were considered statistically significant. The analysis was performed using SPSS software 13.0 (SPSS Inc., Chicago, IL) for windows.

## Results and discussion

In the study, FS was firstly extracted by various solvents to prepare five FS extracts, i.e., petroleum ether extract (PEFS), ethyl acetate extract (EAFS), absolute ethanol extract (AEFS), 95% ethanol extract (95EFS), and water extract (WFS) (Figure 1). Five FS extracts were then determined using an *in vitro* model developed by our laboratory (Li et al. 2013). The results indicated that FS extracts could effectively protect against •OH-induced DNA damage (Additional file 2).

In order to identify which chemical component can be responsible for the protective effect, we further measured the chemical contents in the FS extracts, including total phenolics, total sugars, total saponins, total anthraquinones, aloe-emodin, rhein, and emodin (Additional file 2). On this basis, the correlation graphs (Additional file 3) between chemical contents and the protective effect (1/IC_50_ values, Additional file 2) were plotted to obtain the correlation coefficients (R values). As seen in Table 1, the R values of total phenolics, total sugars and total saponins were respectively 0.769, 0.330, and −0.589. It means that the protective effect of FS may arise from phytophenols, not sugars or saponins. This assumption was further confirmed by the average R values. The average R value of total phenolics was 0.843, while those of total sugars and total saponins were much lower (0.103 and 0.0068 respectively). As we know, phytophenols in FS mainly include phenolic anthraquinones, therefore, total anthraquinones also exhibited a higher R value (0.833, Table 1). Among phenolic anthraquinones, however, aloe-emodin, rhein, and emodin are well-known in FS. In our study, aloe-emodin, rhein, and emodin also possessed higher R values (0.753, 0.820, and 0.784 respectively). Now it is clear that the protective effect against •OH-induced DNA damage of FS can be mainly attributed to phytophenols, especially three phenolic anthraquinones aloe-emodin, rhein, and emodin, not sugars or saponins.

**Table 1:** **The R (correlation coefficient) values between chemical contents and antioxidant levels (1/IC**_**50**_ **value)**

	Total phenolics	Total sugars	Total saponins	Total anthraquinones	Aloe-emodin	Rhein	Emodin
Protection against DNA damage	0.769	0.330	−0.589	0.747	0.773	0.753	0.662
•OH scavenging	0.814	0.561	−0.571	0.650	0.326	0.650	0.575
•O_2_^–^ scavenging	0.865	0.182	0.328	0.743	0.674	0.719	0.664
DPPH• scavenging	0.91	−0.112	0.155	0.997	0.922	0.994	0.983
ABTS^+^• scavenging	0.918	−0.036	0.158	0.962	0.872	0.958	0.931
Cu^2+^-reducing	0.784	−0.306	0.56	0.896	0.952	0.884	0.886
**Average**	**0.843**	**0.103**	**0.0068**	**0.833**	**0.753**	**0.820**	**0.784**

Furthermore, non-enzymatic repair by phytophenols has been reported to be via ROS scavenging and direct DNA radical repairing approaches (Zheng et al. 2010). In order to explore the ROS scavenging possibility of FS, we used our methods (Li 2013; Li 2012) to investigate its ROS scavenging abilities, including •OH-scavenging and •O_2_^–^-scavenging. Our results showed that five FS extracts could eliminate both •OH and •O_2_^–^ radicals (Additional file 2). It suggests that ROS scavenging may play a role in fast non-enzymatic repair of FS.

**Figure 2:**
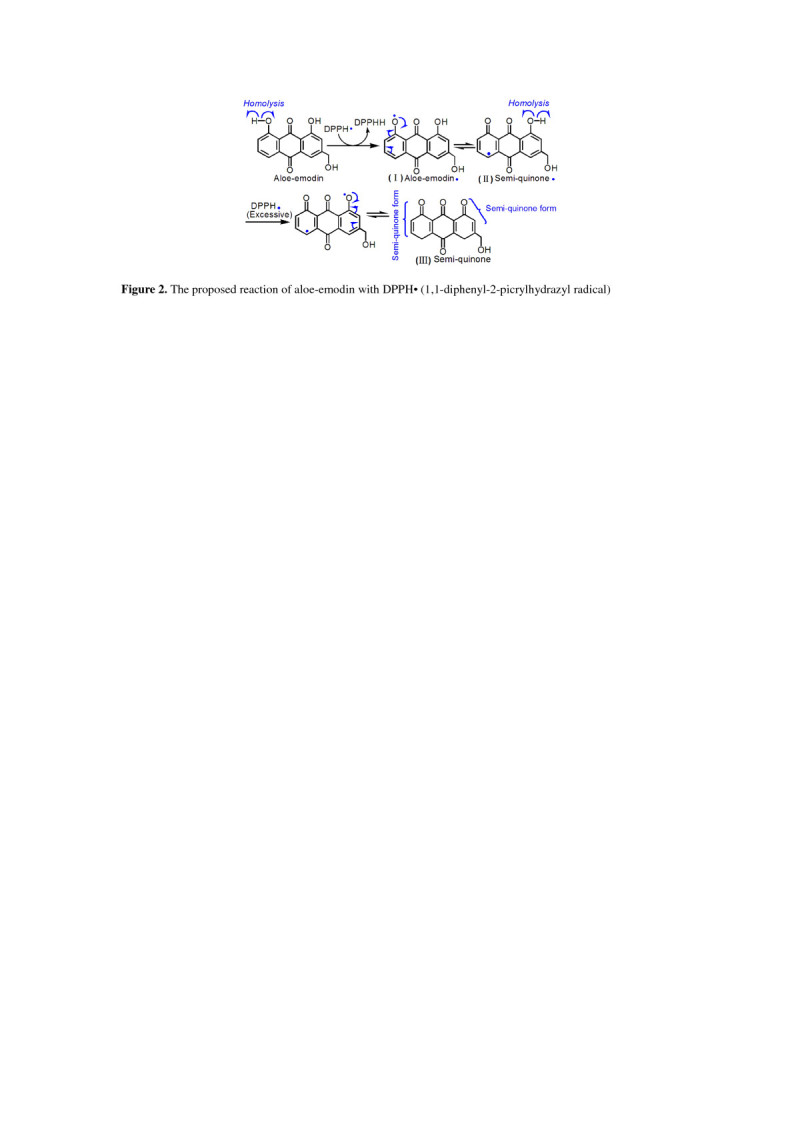
**The proposed reaction of aloe-emodin with DPPH• (1,1-diphenyl-2-picrylhydrazyl radical).**

To study the ROS scavenging mechanism of FS, we determined its radical-scavenging abilities on DPPH• and ABTS^+^•. The data in Additional file 2 indicated that FS could effectively inhibit DPPH• and ABTS^+^• radicals. DPPH• scavenging has been demonstrated to be a hydrogen atom (H•) transfer process (HAT) (Bondet et al. 1997). For example, the proposed reaction for aloe-emodin to scavenge DPPH• can be briefly illustrated in Figure 2. In the process, phenolic –OH underwent homolysis to give H• and aloe-emodin• radical (I). H• was then transferred to DPPH• to generate DPPH-H molecule. Meanwhile, (I) might transform into semi-quinone• radical (II), which could be further extracted H• by excess DPPH• to form the stable semi-quinone (III).

Unlike DPPH• radical, ABTS^+^• radical cation, however, needs only an electron (***e***) to neutralize the positive charge and ABTS^+^• scavenging is regarded as an electron (***e***) transfer process (Aliaga and Lissi 1998). Therefore, in the reaction of aloe-emodin with ABTS^+^• radical, aloe-emodin was thought to produce an electron (***e***) and H^+^ ion. The electron (***e***) was then donated to ABTS^+^• to form stable ABTS molecule. Meanwhile, aloe-emodin changed to the aloe-emodin• radical (I), which could also be converted into semi-quinone• radical (II) and semi-quinone (III) in excess ABTS^+^• (Additional file 4). The electron (***e***) transfer mechanism was also supported by the Cu^2+^-reducing power assay. As we know, reductive reaction is actually an electron (***e***) - donating process. Five FS extracts, however, could successfully reduced Cu^2+^ to Cu^+^ (Additional file 2). Since ***e*** transfer is always accompanied by deprotonation, so it is called sequential electron proton transfer (SEPT) (Iuga et al. 2011).

**Figure 3:**
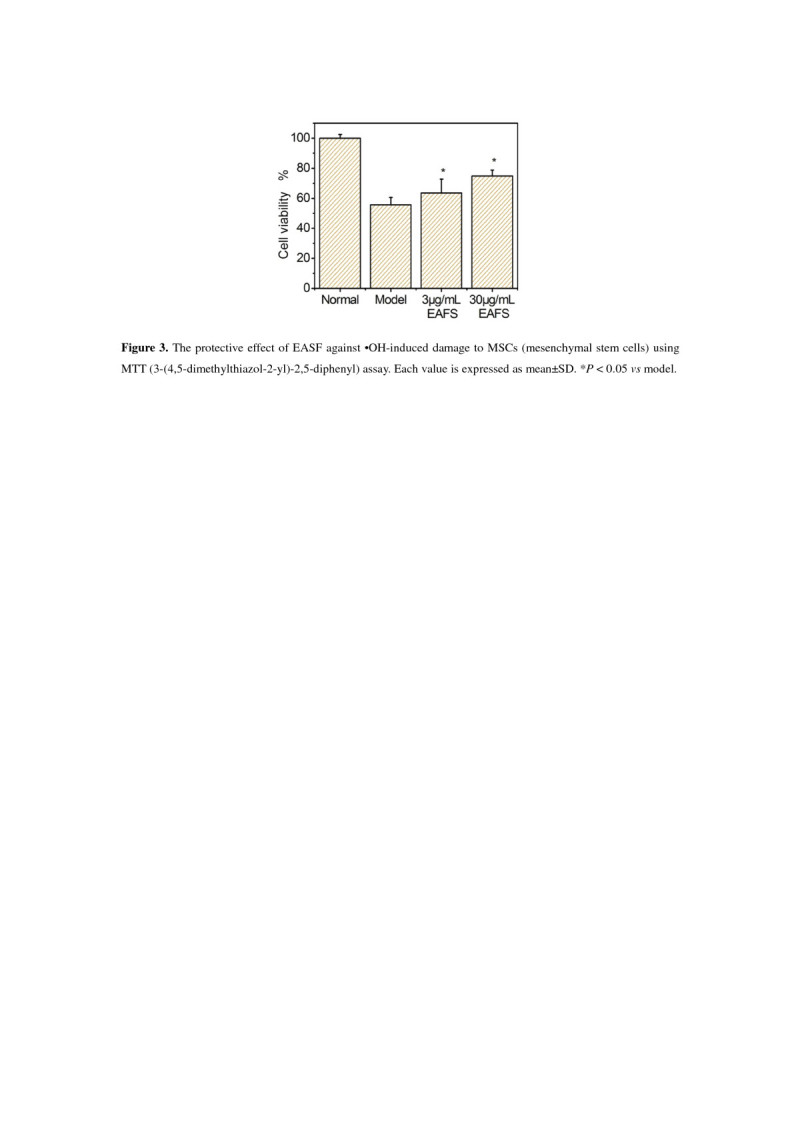
**The protective effect of EAFS against •OH-induced damage to MSCs (mesenchymal stem cells) using MTT (3-(4,5-dimethylthiazol-2-yl)-2,5-diphenyl) assay.** Each value is expressed as mean ± SD, n=3. *P < 0.05 *vs* model.

To obtain biological evidence, the effect of EAFS which has been regarded as the most effective extract among five FS extracts in the antioxidant assays above, was further estimated using the MTT assay. The results suggested that the viability of MSCs has been effectively protected by EAFS (at 3 and 30 μM, Figure 3) when they were treated with •OH radicals. Based on previous reports (Urbanek et al. 2005; Estrada et al. 2013), we assumed that the protective effect against •OH radical-induced damage to MSCs might be directly associated with its repair of oxidative DNA damage.

Taken together, the fact that FS could effectively scavenge both DPPH• and ABTS^+^• radicals, and reduce Cu^2+^, implies that phytophenols in FS exert ROS scavenging action via HAT and/or SEPT mechanisms. Both HAT and SEPT mechanisms, however, can similarly make phytophenols be oxidized to semi-quinone (III). As the final oxidized product, semi-quinone (III) is actually a stable form bearing a large π-π conjugation (Figure 2). Hence, the protective or antioxidant effect of FS may be attributed to the phenolic –OH moiety, and ultimately to the stability of semi-quinone form.

Of course, the HAT and SEPT mechanisms can be used for the interpretation of ROS scavenging. For example, in water at physiological pH 7.4, aloe-emodin could scavenge •OH radical via SEPT mechanism. As we know, carbonyl groups (C = O) can greatly withdraw electron through π-π conjugative systems to enhance the acidity of phenolic –OH groups in aloe-emodin. In the case, the acidity might therefore predominate over its chemical action in the weak alkaline environment, and phenolic –OH would firstly ionize to yield H^+^ ion, and aloe-emodin^-^ which subsequently donated an electron (***e***) to form aloe-emodin• (I) (Additional file 5). However, in the lipidic environment, aloe-emodin scavenged •OH radical via a HAT mechanism: in the case, phenolic –OH in aloe-emodin homolyzed to produce aloe-emodin• radical (I), and a hydrogen atom (H•) which further combined •OH radical to yield H_2_O molecule (Additional file 6). Actually, both possible mechanisms are supported by the previous report (Iuga et al. 2012).

**Figure 4:**
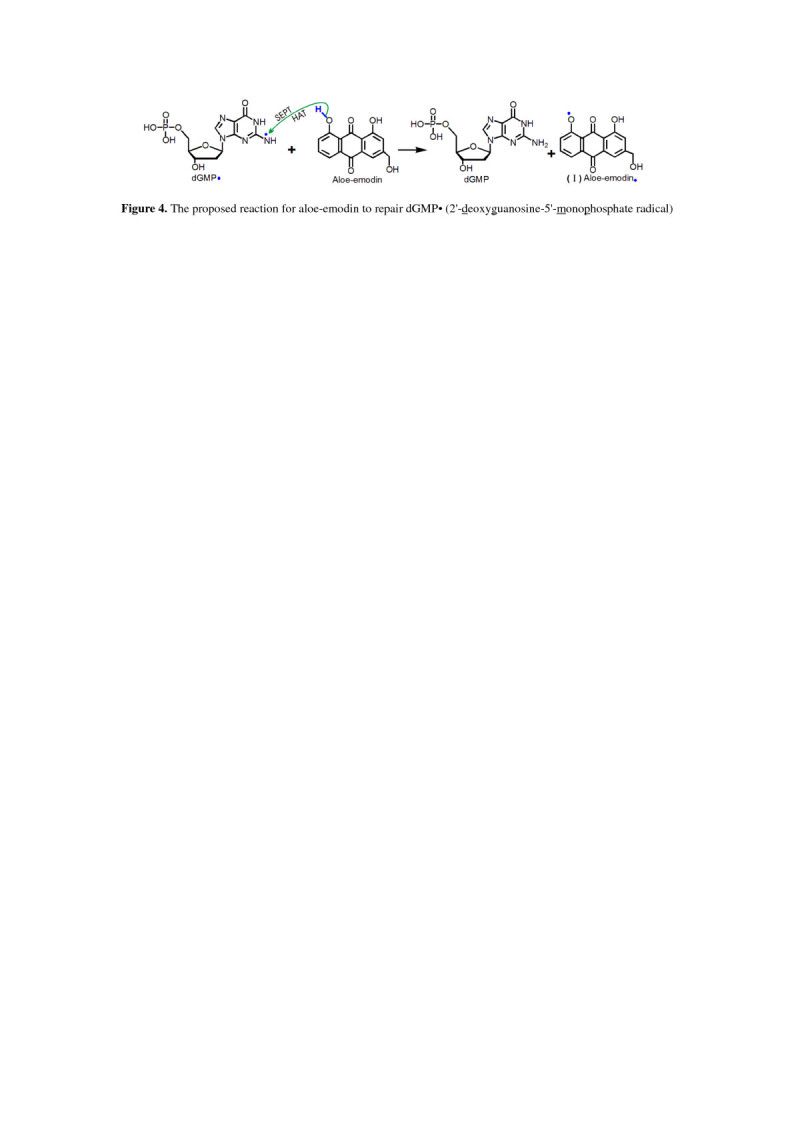
**The proposed reaction for aloe-emodin to repair dGMP• (2′-deoxyguanosine-5′-monophosphate radical).**

More importantly, both SEPT and HAT mechanisms can also be used for the interpretation of direct repairing on DNA radicals, e.g., 2′-deoxyguanosine-5′-monophosphate radical (dGMP•). The dGMP• may be generated via the reaction of nucleotide and •OH radical (Additional file 7). Aloe-emodin, however, could repair dGMP• radical via SEPT and/or HAT mechanisms (Figure 4). As seen in Figure 3, aloe-emodin was oxidized by dGMP• radical to aloe-emodin• radical (I).

## Conclusion

In conclusion, Folium *Sennae* exhibits a protective effect against •OH-induced damages to DNA and MSCs. The effects may be attributed to phytophenols (especially aloe-emodin, rhein, and emodin), not sugars or saponins. They exert the protective action via hydrogen atom transfer (HAT) and/or sequential electron proton transfer (SEPT) mechanisms which make phenolic –OH moiety be oxidized to stable semi-quinone form. The stability of semi-quinone form can ultimately be responsible for the protective or antioxidant effect of phytophenols.

## Electronic supplementary material

Additional file 1:**The photos of Folium** ***Sennae.***(DOC 2 MB)

Additional file 2:**The dose response curves and IC**_**50**_**values of five FS extracts in all assays.**(DOC 1 MB)

Additional file 3:**Correlation graphs.**(DOC 800 KB)

Additional file 4:**The proposed reaction of aloe-emodin with ABTS**^**+**^**•.**(DOC 76 KB)

Additional file 5:**The proposed reaction for aloe-emodin to scavenge •OH via SEPT.**(DOC 45 KB)

Additional file 6:**The proposed reaction for aloe-emodin to scavenge •OH via HAT.**(DOC 40 KB)

Additional file 7:**The proposed reaction of •OH radical attack dGMP to form dGMP•.**(DOC 42 KB)

Below are the links to the authors’ original submitted files for images.

Authors’ original file for figure 1

Authors’ original file for figure 2

Authors’ original file for figure 3

Authors’ original file for figure 4

## Abbreviations

ROS: Reactive oxygen species
HAT: Hydrogen atom transfer
SEPT: Sequential electron proton transfer
FS: Folium *Sennae*
DPPH: 1,1-diphenyl-2-picrylhydrazyl radical
dGMP•: 2′-deoxyguanosine-5′-monophosphate radical
ABTS: 2, 2′-azinobis (3-ethylbenzothiazoline-6-sulfonic acid)
TBA: 2-thiobarbituric acid
PEFS: Petroleum ether extract
EAFS: Ethyl acetate extract
AEFS: Absolute ethanol extract
95EFS: 95% ethanol extract
WFS: Water extract.
