# Supplementary material for: Folium Sennae protects against hydroxyl radical-induced DNA damage via antioxidant mechanism: an in vitro study
Source: Bot Stud. 2014 Feb 2;55:16. doi: 10.1186/1999-3110-55-16 (PMC5430338; doi:10.1186/1999-3110-55-16)
Supplement: Supplementary file 1 — Additional file 1:The photos of Folium Sennae.(DOC 2 MB) [file 40529_2013_68_MOESM1_ESM.doc]

**Additional 1- The photos of Folium Sennae**


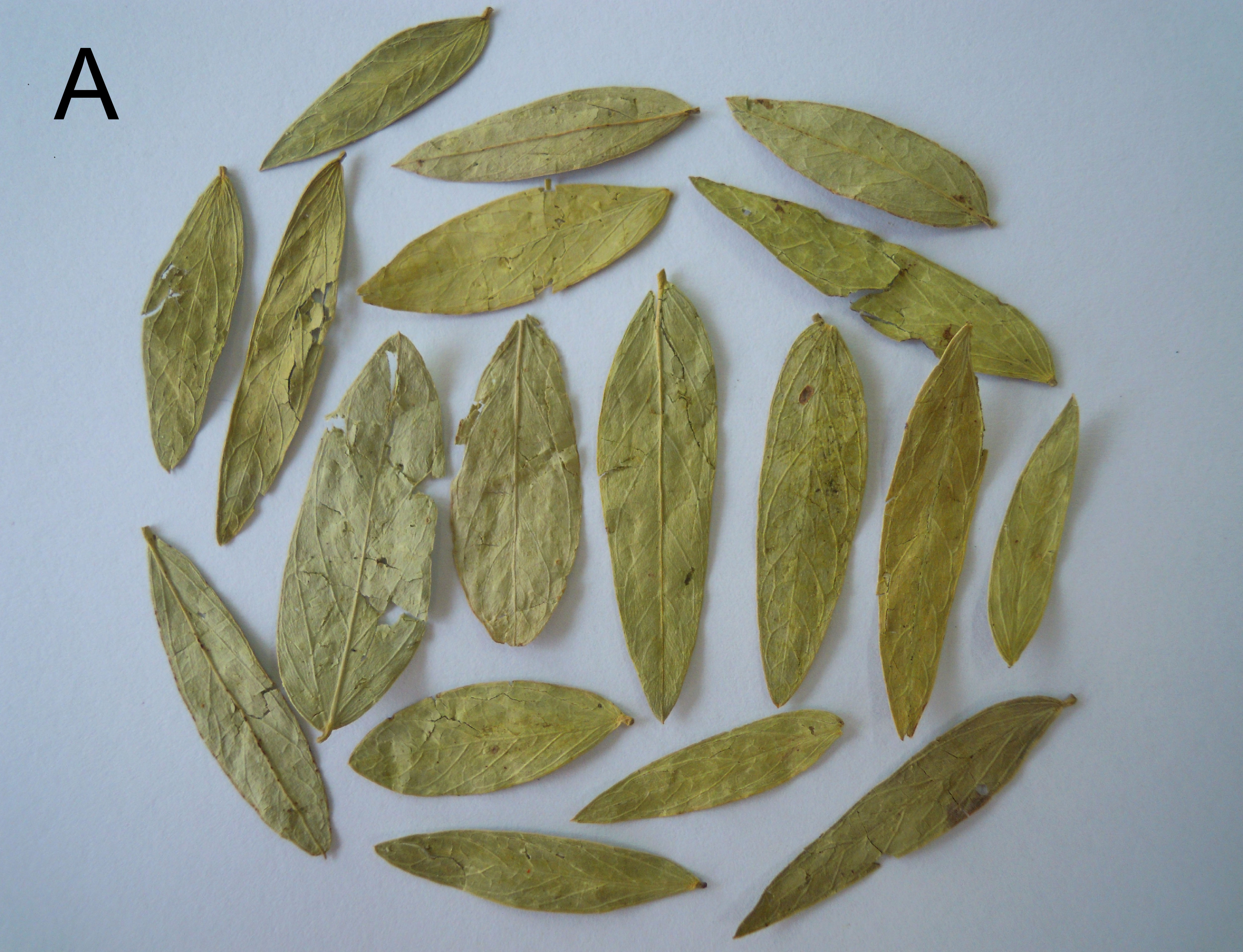

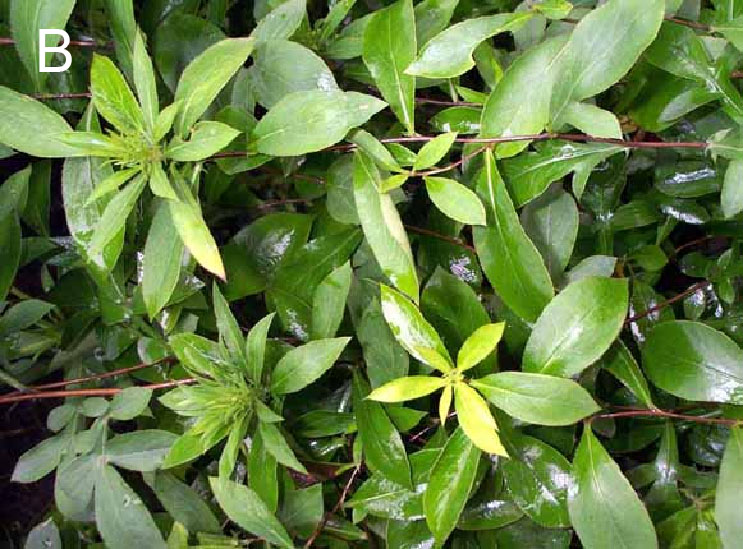


**Figure A1.1** The photos of Folium *Sennae* (the leaves of *Cassia angustifolia* Vahi) (A), and plant of *Cassia angustifolia* Vahi (B)

(Contributed by Jian Lin, in August, 2013)
