# Supplementary material for: Folium Sennae protects against hydroxyl radical-induced DNA damage via antioxidant mechanism: an in vitro study
Source: Bot Stud. 2014 Feb 2;55:16. doi: 10.1186/1999-3110-55-16 (PMC5430338; doi:10.1186/1999-3110-55-16)
Supplement: Supplementary file 2 — Additional file 2:The dose response curves and IC50values of five FS extracts in all assays.(DOC 1 MB) [file 40529_2013_68_MOESM2_ESM.doc]

**Additional 2- The dose response curves and IC_50_ values of five FS extracts in all assays**


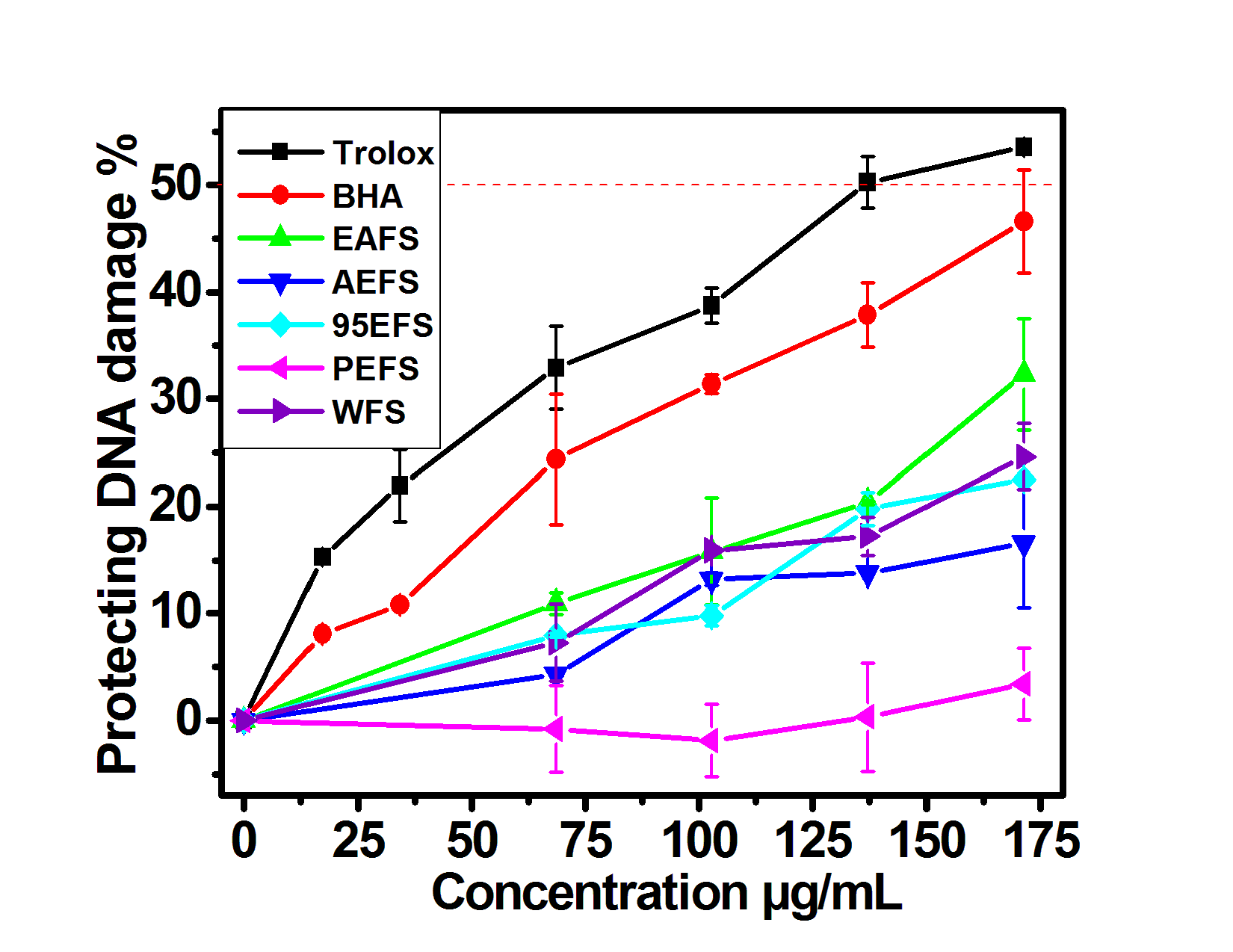


**Figure A2.1** The dose response curves of DNA protective effect (Each value is expressed as Mean±SD, n =3)

PEFS, petroleum ether extract of Folium *Sennae*. EAFS, ethyl acetate extract of Folium *Sennae*. AEFS, absolute ethanol extract of Folium *Sennae*. 95EFS, 95% ethanol extract of Folium *Sennae*. WFS, water extract of Folium *Sennae*.

**Table A2.1** The IC_50_ and 1/IC_50_ values for DNA protective effect

|  | PEFS | EAFS | AEFS | 95EFS | WFS | Trolox | BHA |
| --- | --- | --- | --- | --- | --- | --- | --- |
| IC_50_  (μg/mL) | -- | 228.73±2.34^c^ | 403.20±51.44^d^ | 357.60±3.27^d^ | 350.07±25.20^d^ | 154.60±0.42^a^ | 177.06±5.02^b^ |
| 1/IC_50_  (mL/μg) | -- | 0.00437 | 0.00248 | 0.00280 | 0.00286 |  |  |

IC_50_ value is defined as the concentration of 50% protection percentage. It was calculated by linear regression analysis and expressed as Mean±SD (n=3). The linear regression was analyzed by Origin 6.0 professional software. Means values with different superscripts in the same row are significantly different (*p<*0.05); Means values with same superscripts in the same row are not significantly different (*p<*0.05). PEFS, petroleum ether extract of Folium *Sennae*. EAFS, ethyl acetate extract of Folium *Sennae*. AEFS, absolute ethanol extract of Folium *Sennae*. 95EFS, 95% ethanol extract of Folium *Sennae*. WFS, water extract of Folium *Sennae*. BHA, butylated hydroxyanisole.


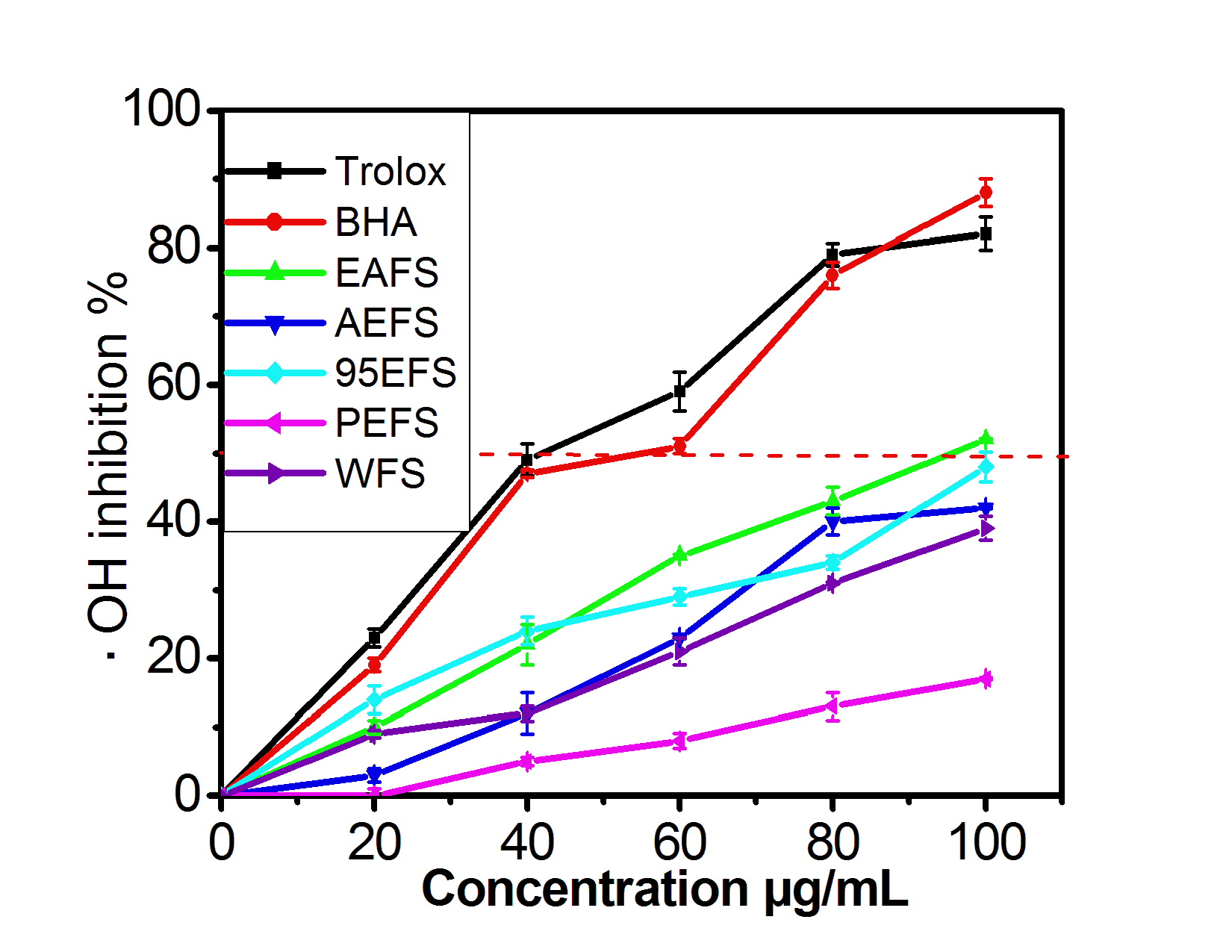


**Figure A2.2** The dose response curves of •OH scavenging activity (Each value is expressed as Mean±SD, n =3)

PEFS, petroleum ether extract of Folium *Sennae*. EAFS, ethyl acetate extract of Folium *Sennae*. AEFS, absolute ethanol extract of Folium *Sennae*. 95EFS, 95% ethanol extract of Folium *Sennae*. WFS, water extract of Folium *Sennae*.

**Table A2.2** The IC_50_ and 1/IC_50_ values for •OH scavenging activity

|  | PEFS | EAFS | AEFS | 95EFS | WFS | Trolox | BHA |
| --- | --- | --- | --- | --- | --- | --- | --- |
| IC_50_  (μg/mL) | 278.36±6.45^g^ | 89.96±3.88 ^c^ | 122.66±6.29^e^ | 99.36±2.55 ^d^ | 132.56±3.87^f^ | 40.66±1.58 ^a^ | 43.26±1.03^b^ |
| 1/IC_50_  (mL/μg) | 0.0036 | 0.011 | 0.0082 | 0.010 | 0.0075 |  |  |

IC_50_ value is defined as the concentration of 50% protection percentage. It was calculated by linear regression analysis and expressed as Mean±SD (n=3). The linear regression was analyzed by Origin 6.0 professional software. Means values with different superscripts in the same row are significantly different (*p<*0.05); Means values with same superscripts in the same row are not significantly different (*p<*0.05). PEFS, petroleum ether extract of Folium *Sennae*. EAFS, ethyl acetate extract of Folium *Sennae*. AEFS, absolute ethanol extract of Folium *Sennae*. 95EFS, 95% ethanol extract of Folium *Sennae*. WFS, water extract of Folium *Sennae*. BHA, butylated hydroxyanisole.


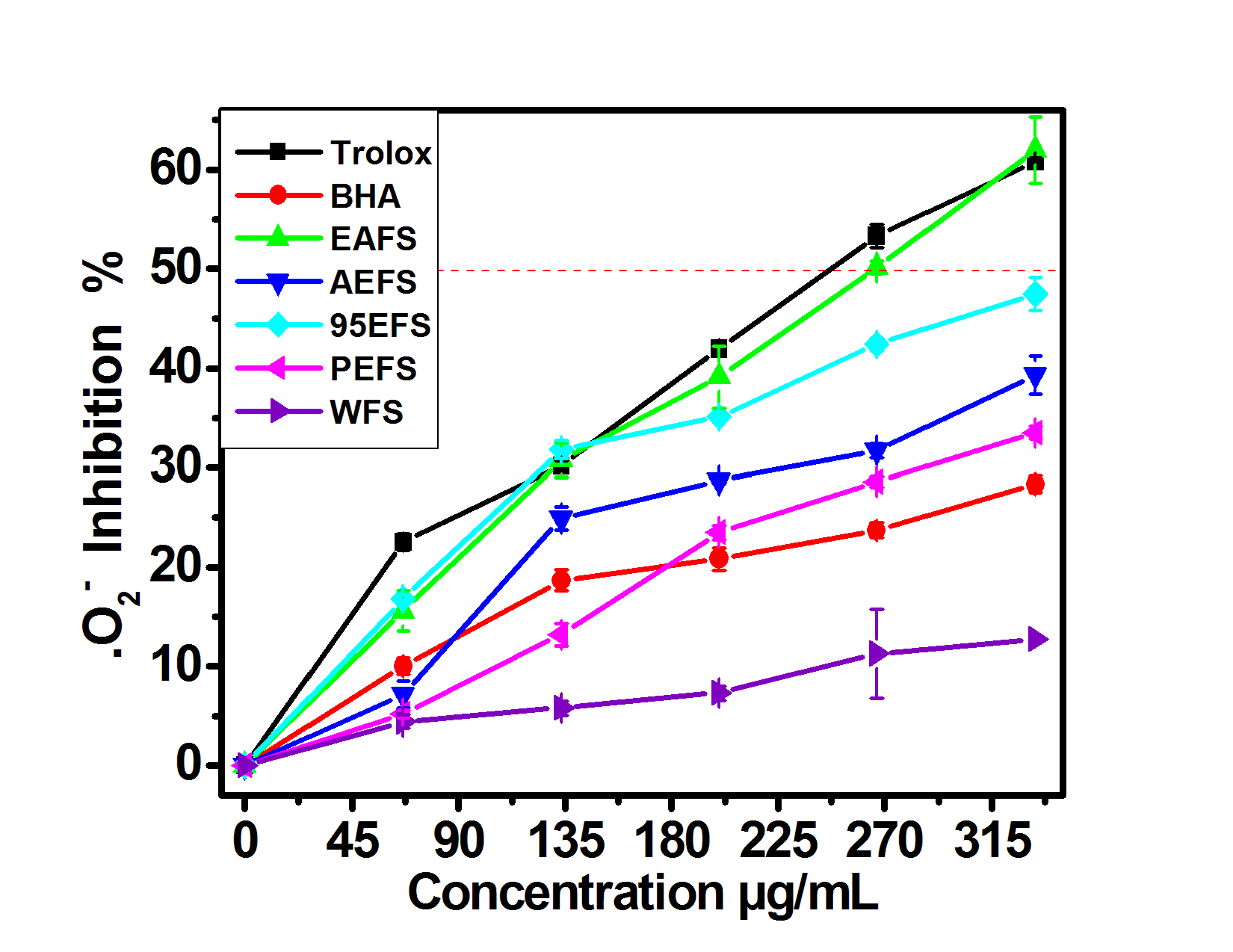


**Figure A2.3** The dose response curves of •**O_2_ -** scavenging activity (Each value is expressed as Mean±SD, n =3)

PEFS, petroleum ether extract of Folium *Sennae*. EAFS, ethyl acetate extract of Folium *Sennae*. AEFS, absolute ethanol extract of Folium *Sennae*. 95EFS, 95% ethanol extract of Folium *Sennae*. WFS, water extract of Folium *Sennae*.

**Table A2.3** The IC_50_ and 1/IC_50_ values for •**O_2_ -** scavenging activity

|  | PEFS | EAFS | AEFS | 95EFS | WFS | Trolox | BHA |
| --- | --- | --- | --- | --- | --- | --- | --- |
| IC_50_  (μg/mL) | 479.37±3.90^d^ | 260.10±7.27^a^ | 427.34±5.30^c^ | 337.00±5.07^b^ | 2646.66± 194.42^f^ | 253.96±2.26^a^ | 649.62±6.49^e^ |
| 1/IC_50_  (mL/μg) | 0.00209 | 0.00384 | 0.00234 | 0.00297 | 0.00038 |  |  |

IC_50_ value is defined as the concentration of 50% protection percentage. It was calculated by linear regression analysis and expressed as Mean±SD (n=3). The linear regression was analyzed by Origin 6.0 professional software. Means values with different superscripts in the same row are significantly different (*p<*0.05); Means values with same superscripts in the same row are not significantly different (*p<*0.05). PEFS, petroleum ether extract of Folium *Sennae*. EAFS, ethyl acetate extract of Folium *Sennae*. AEFS, absolute ethanol extract of Folium *Sennae*. 95EFS, 95% ethanol extract of Folium *Sennae*. WFS, water extract of Folium *Sennae*. BHA, butylated hydroxyanisole.


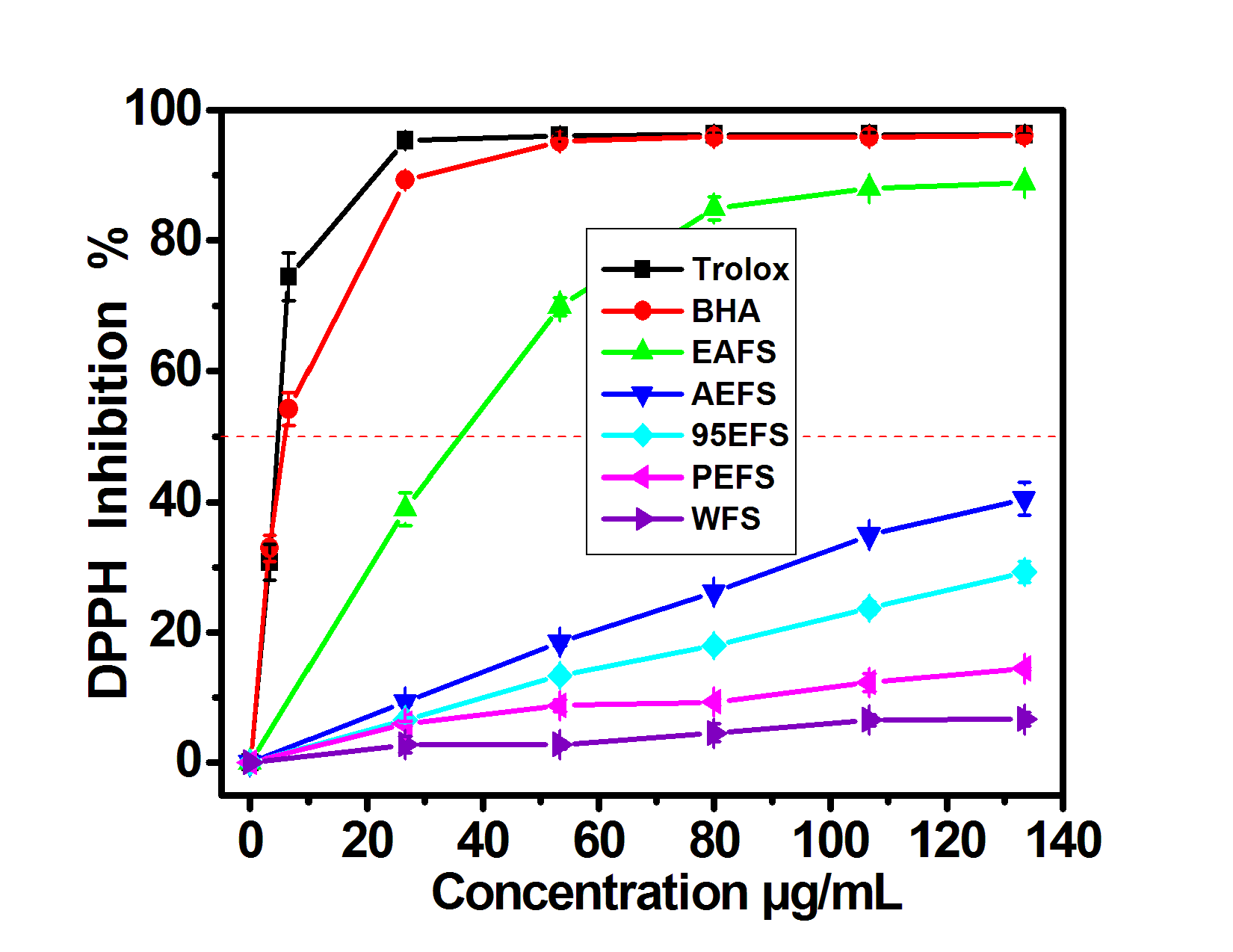


**Figure A2.4** The dose response curves of DPPH• scavenging activity (Each value is expressed as Mean±SD, n =3)

PEFS, petroleum ether extract of Folium *Sennae*. EAFS, ethyl acetate extract of Folium *Sennae*. AEFS, absolute ethanol extract of Folium *Sennae*. 95EFS, 95% ethanol extract of Folium *Sennae*. WFS, water extract of Folium *Sennae*.

**Table A2.4** The IC_50_ and 1/IC_50_ values for DPPH• scavenging activity

|  | PEFS | EAFS | AEFS | 95EFS | WFS | Trolox | BHA |
| --- | --- | --- | --- | --- | --- | --- | --- |
| IC_50_  (μg/mL) | 550.83±15.23^f^ | 36.40±2.31^c^ | 158.00±0.30^d^ | 232.27±10.40^e^ | 750.71±61.35^g^ | 4.15±0.80^a^ | 8.33±0.32^b^ |
| 1/IC_50_  (mL/μg) | 0.00182 | 0.02747 | 0.00633 | 0.00431 | 0.00133 |  |  |

IC_50_ value is defined as the concentration of 50% protection percentage. It was calculated by linear regression analysis and expressed as Mean±SD (n=3). The linear regression was analyzed by Origin 6.0 professional software. Means values with different superscripts in the same row are significantly different (*p<*0.05); Means values with same superscripts in the same row are not significantly different (*p<*0.05). PEFS, petroleum ether extract of Folium *Sennae*. EAFS, ethyl acetate extract of Folium *Sennae*. AEFS, absolute ethanol extract of Folium *Sennae*. 95EFS, 95% ethanol extract of Folium *Sennae*. WFS, water extract of Folium *Sennae*. BHA, butylated hydroxyanisole.


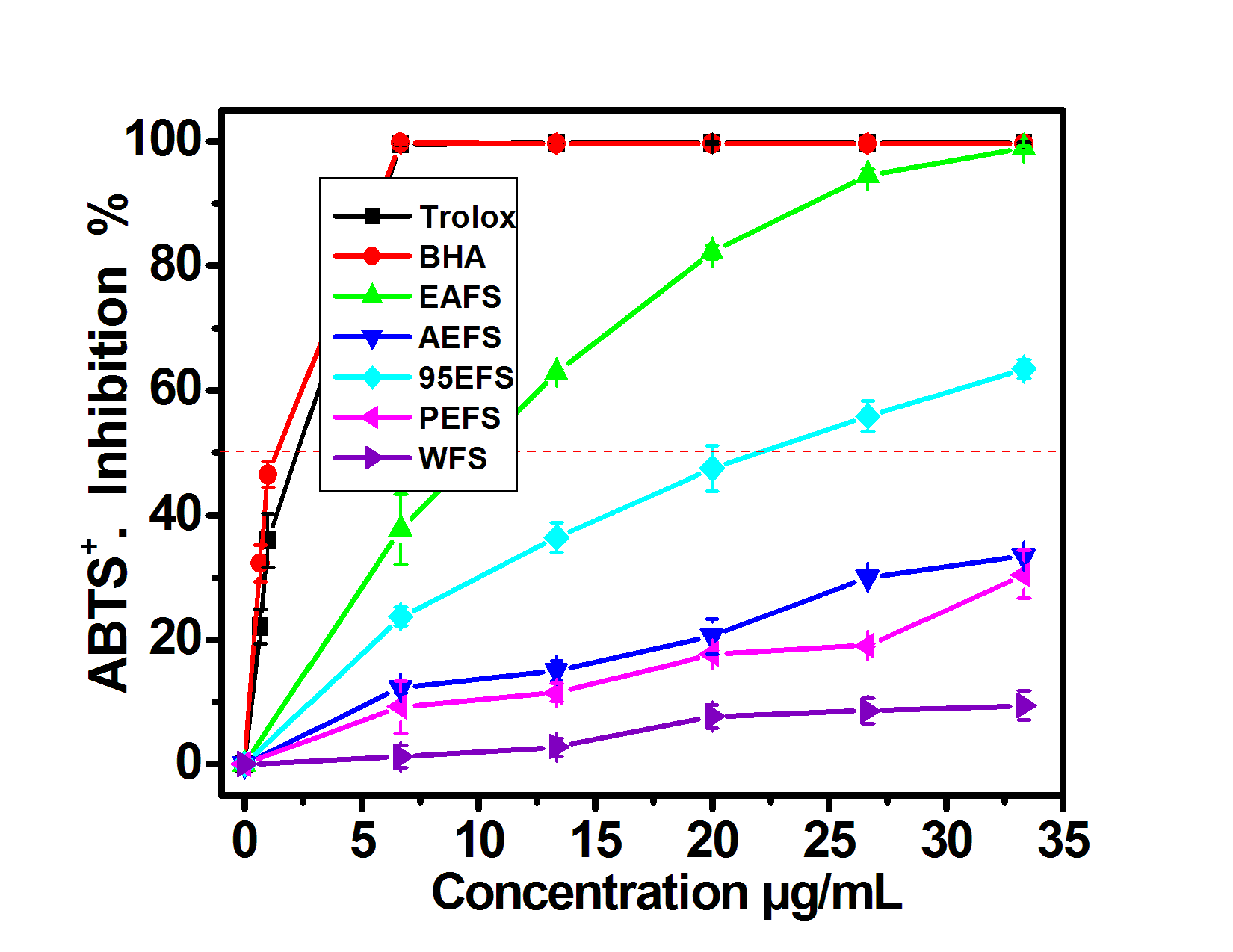


**Figure A2.5** The dose response curves of ABTS^+^• scavenging activity (Each value is expressed as Mean±SD, n =3)

PEFS, petroleum ether extract of Folium *Sennae*. EAFS, ethyl acetate extract of Folium *Sennae*. AEFS, absolute ethanol extract of Folium *Sennae*. 95EFS, 95% ethanol extract of Folium *Sennae*. WFS, water extract of Folium *Sennae*.

**Table A2.5** The IC_50_ and 1/IC_50_ values for ABTS^+^• scavenging activity

|  | PEFS | EAFS | AEFS | 95EFS | WFS | Trolox | BHA |
| --- | --- | --- | --- | --- | --- | --- | --- |
| IC_50_  (μg/mL) | 60.74±2.43^f^ | 10.35±0.51^c^ | 51.99±0.91^e^ | 23.47±0.45^d^ | 149.17±16.18^g^ | 2.58±0.03^b^ | 1.93±0.07^a^ |
| 1/IC_50_  (mL/μg) | 0.01646 | 0.09663 | 0.01924 | 0.04261 | 0.00670 |  |  |

IC_50_ value is defined as the concentration of 50% protection percentage. It was calculated by linear regression analysis and expressed as Mean±SD (n=3). The linear regression was analyzed by Origin 6.0 professional software. Means values with different superscripts in the same row are significantly different (*p<*0.05); Means values with same superscripts in the same row are not significantly different (*p<*0.05). PEFS, petroleum ether extract of Folium *Sennae*. EAFS, ethyl acetate extract of Folium *Sennae*. AEFS, absolute ethanol extract of Folium *Sennae*. 95EFS, 95% ethanol extract of Folium *Sennae*. WFS, water extract of Folium *Sennae*. BHA, butylated hydroxyanisole.


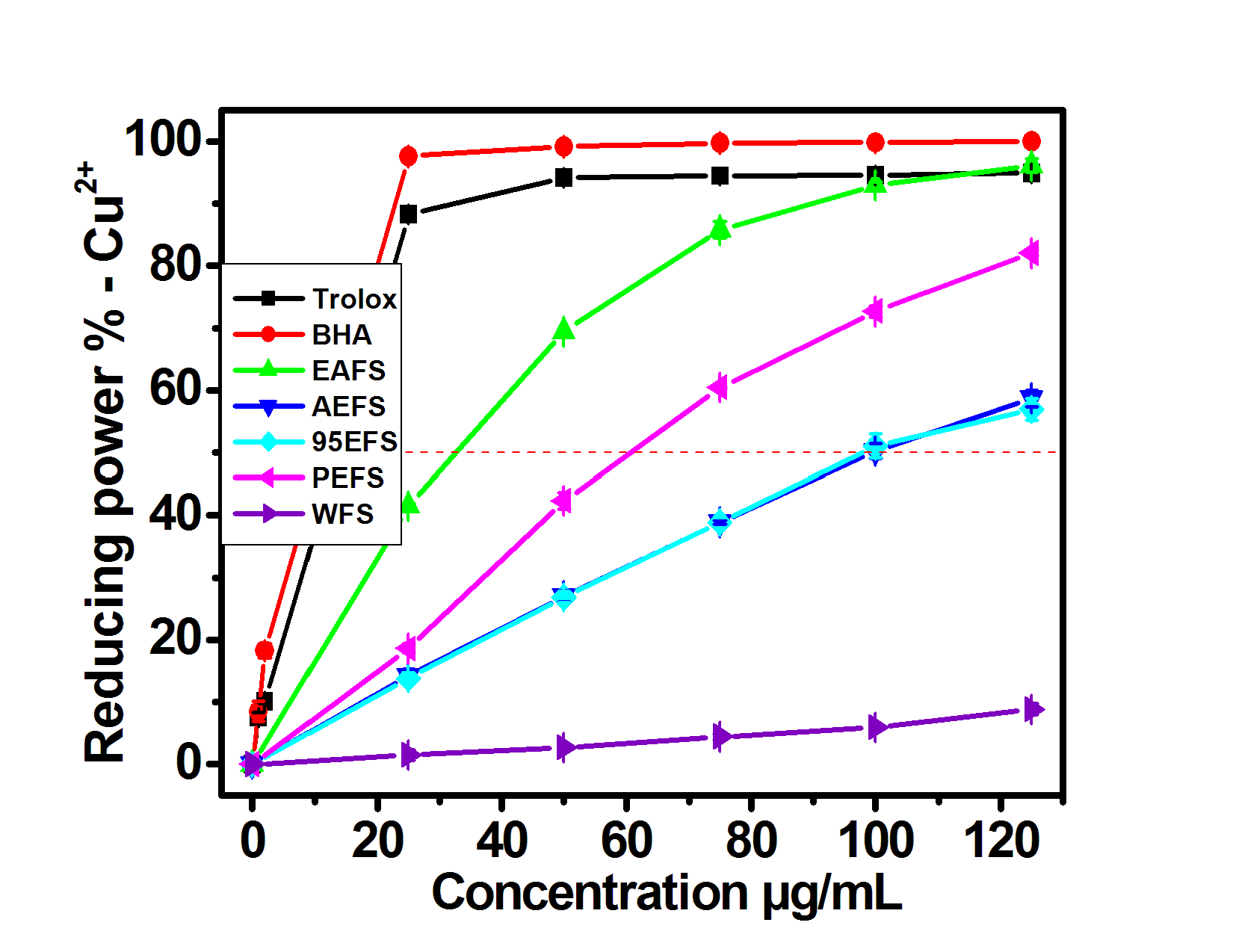


**Figure A2.6** The dose response curves of Cu^2+^ reducing power assay (Each value is expressed as Mean±SD, n =3)

PEFS, petroleum ether extract of Folium *Sennae*. EAFS, ethyl acetate extract of Folium *Sennae*. AEFS, absolute ethanol extract of Folium *Sennae*. 95EFS, 95% ethanol extract of Folium *Sennae*. WFS, water extract of Folium *Sennae*.

**Table A2.6** The IC_50_ and 1/IC_50_ values for Cu^2+^ reducing power

|  | PEFS | EAFS | AEFS | 95EFS | WFS | Trolox | BHA |
| --- | --- | --- | --- | --- | --- | --- | --- |
| IC_50_  (μg/mL) | 64.56±0.41^d^ | 32.38±0.33^c^ | 98.73±1.76^e^ | 97.60±2.76^e^ | 694.29±28.28^f^ | 13.69±0.06^b^ | 11.65±0.09^a^ |
| 1/IC_50_  (mL/μg) | 0.01549 | 0.03088 | 0.01013 | 0.01025 | 0.00144 |  |  |

IC_50_ value is defined as the concentration of 50% protection percentage. It was calculated by linear regression analysis and expressed as Mean±SD (n=3). The linear regression was analyzed by Origin 6.0 professional software. Means values with different superscripts in the same row are significantly different (*p<*0.05); Means values with same superscripts in the same row are not significantly different (*p<*0.05). PEFS, petroleum ether extract of Folium *Sennae*. EAFS, ethyl acetate extract of Folium *Sennae*. AEFS, absolute ethanol extract of Folium *Sennae*. 95EFS, 95% ethanol extract of Folium *Sennae*. WFS, water extract of Folium *Sennae*. BHA, butylated hydroxyanisole.


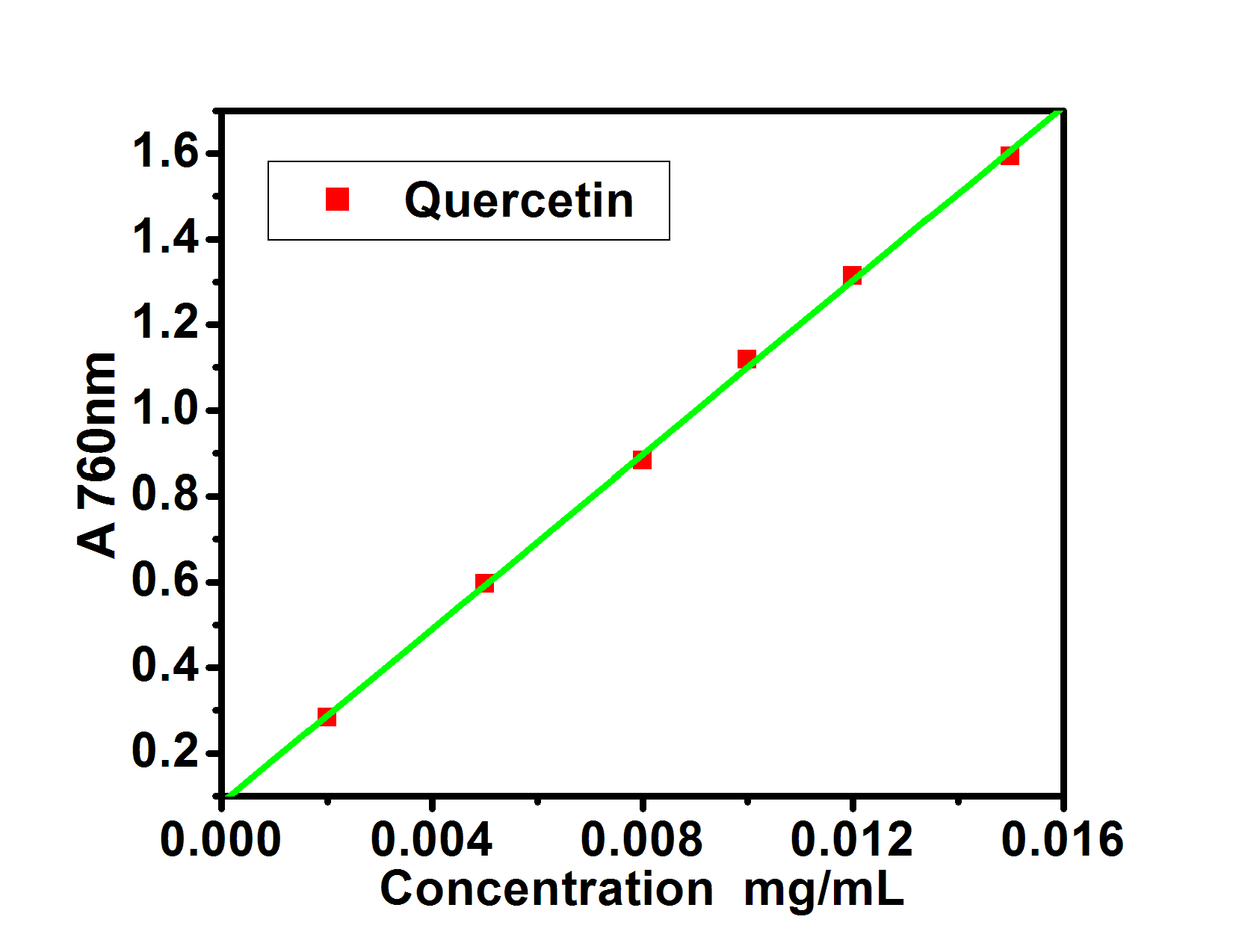


**Figure A2.7** The standard curve of quercetin (*y* = 101.53*x* + 0.0842, R = 0.9996)

**Table A2.7** The total phenolics contents of five extracts from Folium *Sennae*

|  | PEFS | EAFS | AEFS | 95EFS | WFS |
| --- | --- | --- | --- | --- | --- |
| A760nm | 0.121 | 0.7 | 0.389 | 0.376 | 0.101 |
|  | 0.127 | 0.701 | 0.375 | 0.372 | 0.1 |
|  | 0.12 | 0.699 | 0.389 | 0.371 | 0.1 |
| Content  (mg quercetin/g) | 3.625 | 121.304 | 60.042 | 57.48 | 1.655 |
|  | 4.216 | 121.502 | 57.284 | 56.692 | 1.556 |
|  | 3.526 | 121.108 | 60.042 | 56.496 | 1.556 |
| Mean±SD | 3.79±0.37^b^ | 121.30±0.20^d^ | 59.12±1.59^c^ | 56.89±0.52^c^ | 1.59±0.06^a^ |

PEFS, petroleum ether extract of Folium *Sennae*. EAFS, ethyl acetate extract of Folium *Sennae*. AEFS, absolute ethanol extract of Folium *Sennae*. 95EFS, 95% ethanol extract of Folium *Sennae*. WFS, water extract of Folium *Sennae*.


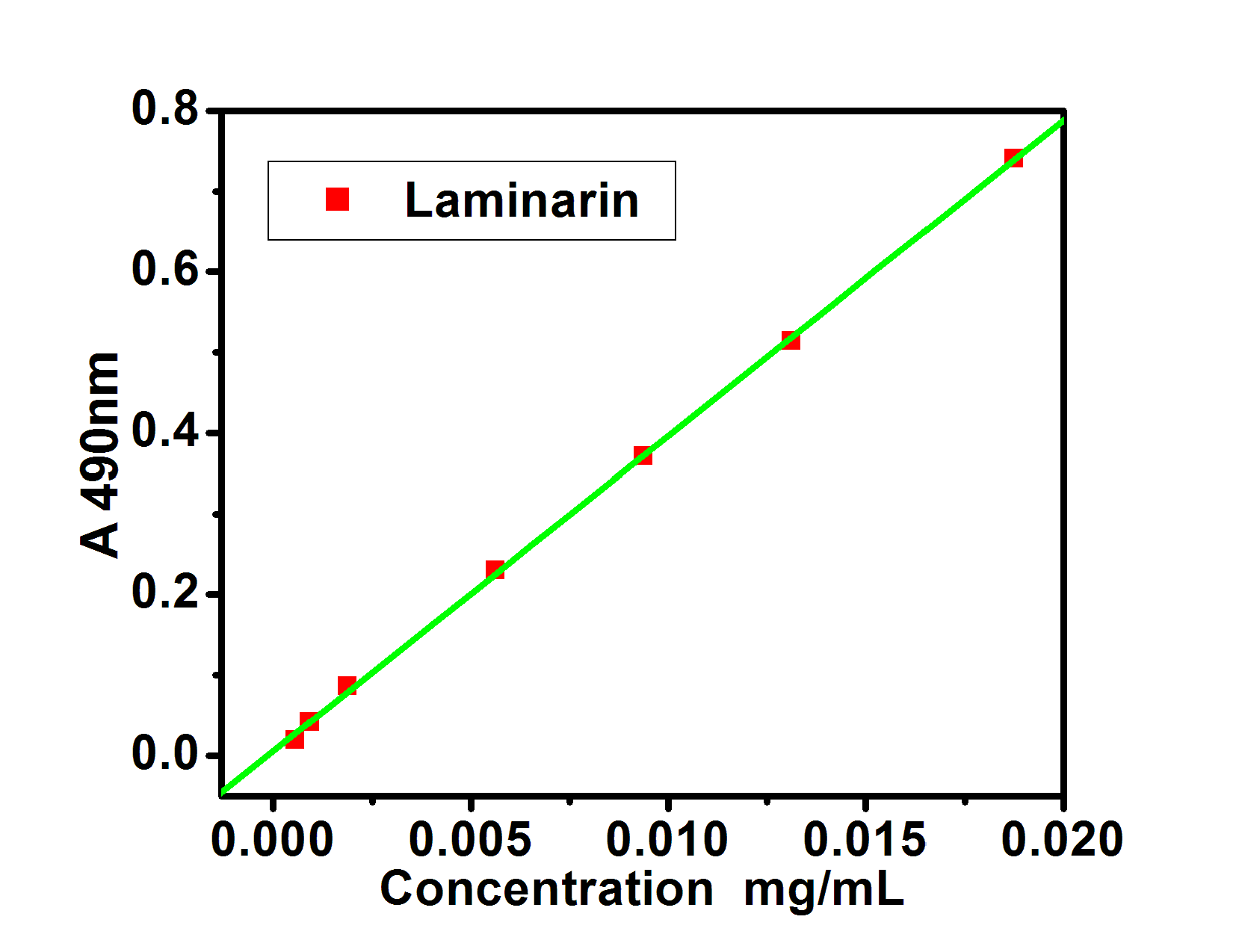


**Figure A2.8** The standard curve of laminarin (*y* = 39.098*x* + 0.0054, R = 0.9998)

**Table A2.8** The total sugars contents of five extracts from Folium *Sennae*

|  | PEFS | EAFS | AEFS | 95EFS | WFS |
| --- | --- | --- | --- | --- | --- |
| A490nm | 0.063 | 0.171 | 0.43 | 0.431 | 0.27 |
|  | 0.039 | 0.158 | 0.421 | 0.41 | 0.296 |
|  | 0.065 | 0.171 | 0.427 | 0.431 | 0.27 |
| Content  (mg laminarin/g) | 39.285333 | 169.42 | 434.396 | 435.42 | 180.46933 |
|  | 22.917333 | 156.12 | 425.188 | 413.936 | 198.20267 |
|  | 40.650667 | 169.42 | 431.328 | 435.42 | 180.46933 |
| Mean±SD | 34.28±9.87^a^ | 164.99±7.68^b^ | 430.30±4.69^d^ | 428.26±12.40^d^ | 186.38±10.24^c^ |

PEFS, petroleum ether extract of Folium *Sennae*. EAFS, ethyl acetate extract of Folium *Sennae*. AEFS, absolute ethanol extract of Folium *Sennae*. 95EFS, 95% ethanol extract of Folium *Sennae*. WFS, water extract of Folium *Sennae*.


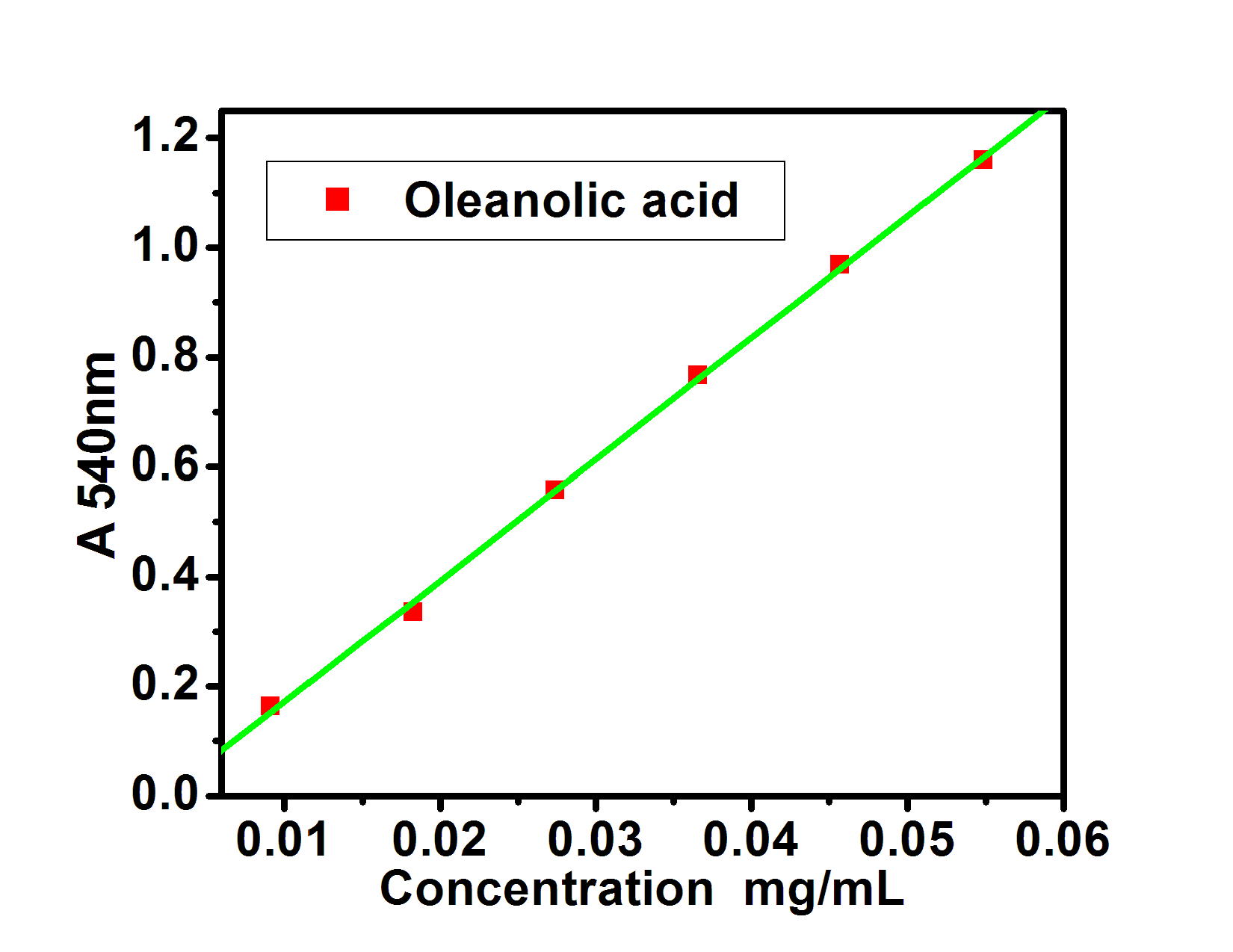


**Figure A2.9** The standard curve of oleanolic acid (*y* = 22.134*x* - 0.0498, R = 0.9996)

***Table A2.9*** *The total saponins contents of five extracts from Folium* Sennae

|  | PEFS | EAFS | AEFS | 95EFS | WFS |
| --- | --- | --- | --- | --- | --- |
| A540nm | 0.742 | 0.586 | 0.262 | 0.42 | 0.035 |
|  | 0.74 | 0.649 | 0.289 | 0.472 | 0.031 |
|  | 0.741 | 0.609 | 0.281 | 0.453 | 0.033 |
| Content  (mg oleanolic acid/g) | 313.0138 | 167.5625 | 82.1736 | 61.9071 | 22.3487 |
|  | 312.2236 | 184.1659 | 89.2897 | 68.7593 | 21.2946 |
|  | 312.6183 | 173.6245 | 87.1809 | 66.2556 | 21.8219 |
| Mean±SD | 312.62±0.40^e^ | 175.12±8.40^d^ | 86.21±3.66^c^ | 65.64±3.47^b^ | 21.82±0.53^a^ |

PEFS, petroleum ether extract of Folium *Sennae*. EAFS, ethyl acetate extract of Folium *Sennae*. AEFS, absolute ethanol extract of Folium *Sennae*. 95EFS, 95% ethanol extract of Folium *Sennae*. WFS, water extract of Folium *Sennae*.


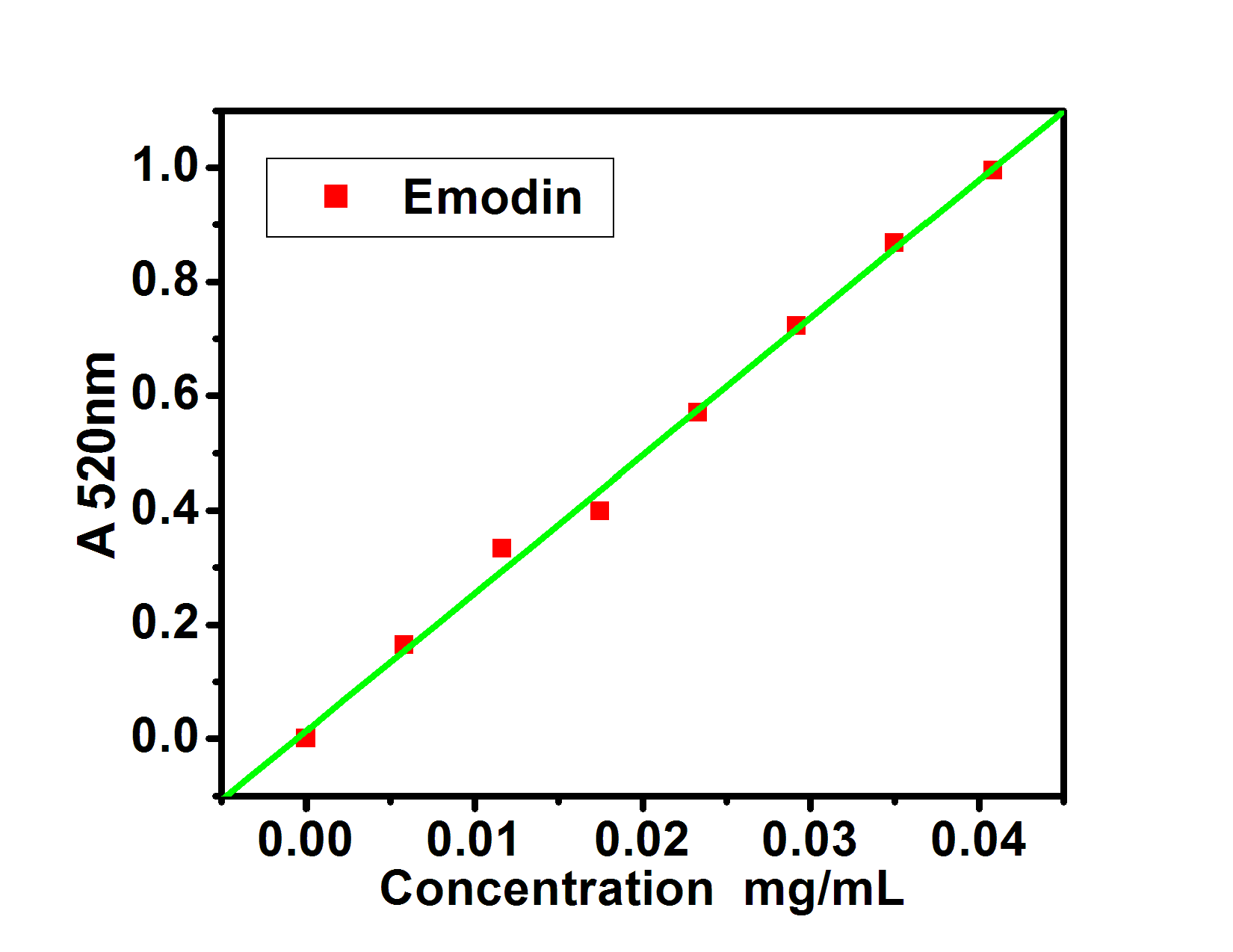


**Figure A2.10** The standard curve of emodin (*y* = 24.127*x* + 0.0136, R = 0.998)

**Table A2.10** The total anthraquinones contents of five extracts from Folium *Sennae*

|  | PEFS | EAFS | AEFS | 95EFS | WFS |
| --- | --- | --- | --- | --- | --- |
| A520nm | 0.073 | 0.814 | 0.172 | 0.198 | 0.071 |
|  | 0.083 | 0.874 | 0.195 | 0.186 | 0.058 |
|  | 0.051 | 0.815 | 0.196 | 0.187 | 0.064 |
| Content  (mg emodin/g) | 9.2325 | 124.40438 | 19.6959 | 22.9287 | 7.1373 |
|  | 10.7865 | 133.72988 | 22.5555 | 21.4365 | 5.5209 |
|  | 5.812875 | 124.55963 | 22.68 | 21.561 | 6.2667 |
| Mean±SD | 8.61±2.54^a^ | 127.56±5.34^c^ | 21.64±1.69^b^ | 21.98±0.83^b^ | 6.31±0.81^a^ |

PEFS, petroleum ether extract of Folium *Sennae*. EAFS, ethyl acetate extract of Folium *Sennae*. AEFS, absolute ethanol extract of Folium *Sennae*. 95EFS, 95% ethanol extract of Folium *Sennae*. WFS, water extract of Folium *Sennae*.


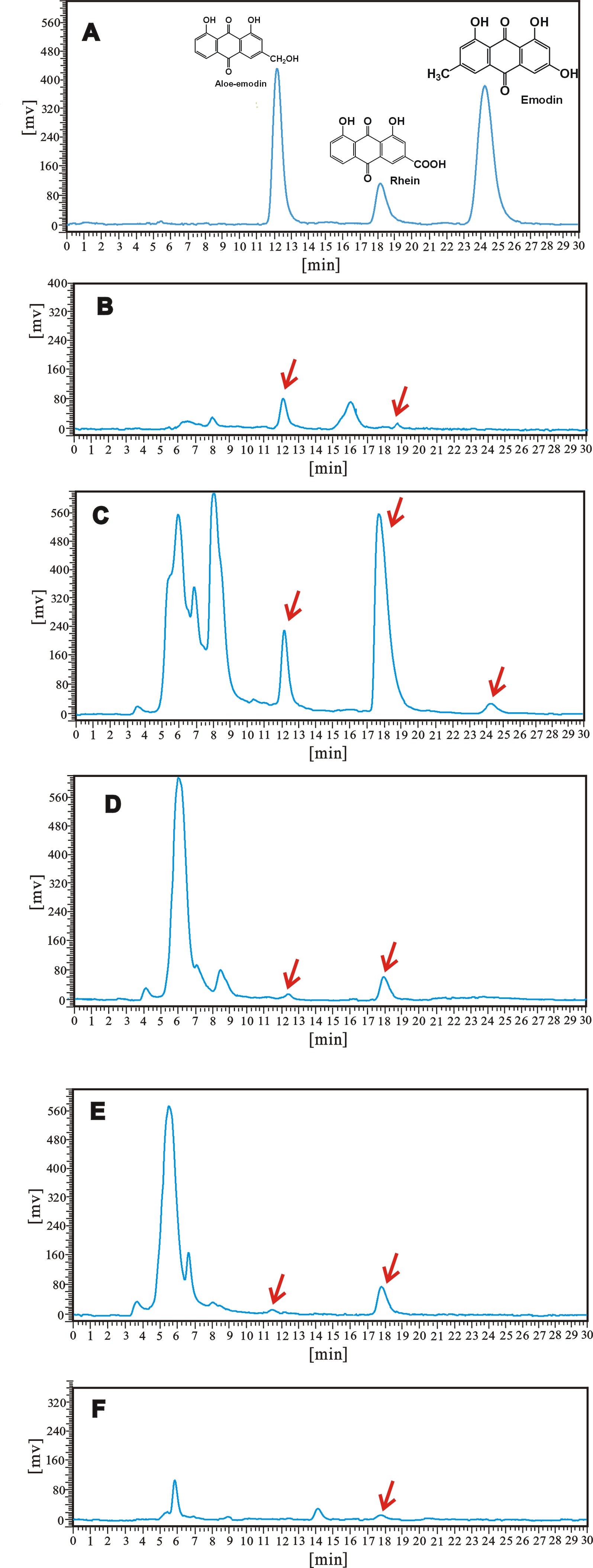


**Figure A2.11** Typical HPLC profiles of (A) standard aloe-emodin, rhein and emodin; (B) petroleum ether extract from Folium *Sennae* (PEFS); (C) ethyl acetate extract from Folium *Sennae* (EAFS); (D) absolute ethanol extract from Folium *Sennae* (AEFS); (E) 95% ethanol extract from Folium *Sennae* (95EFS); and (F) water extract from Folium *Sennae* (WFS).

**Table A2.11 The retention time, peak area of aloe-emodin, rhein and emodin in five extracts from Folium *Sennae***

|  | Compound | Retention time/min | Peak area * |
| --- | --- | --- | --- |
| PEFS | aloe-emodin | 12.165 | 2890158.25 ± 51478.02 |
|  | rhein | 18.032 | 242645.77 ± 18933.57 |
|  | emodin | -- | 0 |
| EAFS | aloe-emodin | 12.115 | 8470492.50 ± 493711.04 |
|  | rhein | 18.132 | 31018420.00 ± 308724.65 |
|  | emodin | 23.748 | 1547685.33 ± 50294.75 |
| AEFS | aloe-emodin | 12.235 | 769952.23 ± 52224.38 |
|  | rhein | 18.012 | 3021922.58 ± 136222.39 |
|  | emodin | -- | 0 |
| 95EFS | aloe-emodin | 11.598 | 486443.86 ± 10512.65 |
|  | rhein | 17.965 | 3718584.83 ± 194437.60 |
|  | emodin | -- | 0 |
| WFS | aloe-emodin | -- | 0 |
|  | rhein | 18.065 | 651921.71 ± 21373.32 |
|  | emodin | -- | 0 |

* The values are expressed as Mean±SD (*n*=3). PEFS, petroleum ether extract of Folium *Sennae*. EAFS, ethyl acetate extract of Folium *Sennae*. AEFS, absolute ethanol extract of Folium *Sennae*. 95EFS, 95% ethanol extract of Folium *Sennae*. WFS, water extract of Folium *Sennae*.
