# Supplementary material for: Folium Sennae protects against hydroxyl radical-induced DNA damage via antioxidant mechanism: an in vitro study
Source: Bot Stud. 2014 Feb 2;55:16. doi: 10.1186/1999-3110-55-16 (PMC5430338; doi:10.1186/1999-3110-55-16)
Supplement: Supplementary file 3 — Additional file 3:Correlation graphs.(DOC 800 KB) [file 40529_2013_68_MOESM3_ESM.doc]

**Additional 3- Correlation graphs**

**Figure A2.1** The correlation graphs between **protective effect** against DNA damage and chemical contents: (A) for total phenolics; (B) for total sugars; (C) for total saponins; (D) for total anthraquinones; (E) for aloe-emodin; (F) for rhein; (G) for emodin.

**Figure A2.2** The correlation graphs between **•OH scavenging** activity and chemical contents: (A) for total phenolics; (B) for total sugars; (C) for total saponins; (D) for total anthraquinones; (E) for aloe-emodin; (F) for rhein; (G) for emodin.

**Figure A2.3** The correlation graphs between **•O_2_^−^ scavenging** activity and chemical contents: (A) for total phenolics; (B) for total sugars; (C) for total saponins; (D) for total anthraquinones; (E) for aloe-emodin; (F) for rhein; (G) for emodin.

**Figure A2.4** The correlation graphs between **DPPH• scavenging** activity and chemical contents: (A) for total phenolics; (B) for total sugars; (C) for total saponins; (D) for total anthraquinones; (E) for aloe-emodin; (F) for rhein; (G) for emodin.

**Figure A2.5** The correlation graphs between **ABTS^+^• scavenging** activity and chemical contents: (A) for total phenolics; (B) for total sugars; (C) for total saponins; (D) for total anthraquinones; (E) for aloe-emodin; (F) for rhein; (G) for emodin.

**Figure A2.6** The correlation graphs between **Cu^2+^ reducing power** and chemical contents: (A) for total phenolics; (B) for total sugars; (C) for total saponins; (D) for total anthraquinones; (E) for aloe-emodin; (F) for rhein; (G) for emodin.
