# Supplementary material for: Folium Sennae protects against hydroxyl radical-induced DNA damage via antioxidant mechanism: an in vitro study
Source: Bot Stud. 2014 Feb 2;55:16. doi: 10.1186/1999-3110-55-16 (PMC5430338; doi:10.1186/1999-3110-55-16)
Supplement: Supplementary file 4 — Additional file 4:The proposed reaction of aloe-emodin with ABTS+•.(DOC 76 KB) [file 40529_2013_68_MOESM4_ESM.doc]

**Additional 4-The proposed reaction of aloe-emodin with ABTS^+^**•
