# Supplementary figures and images for: Folium Sennae protects against hydroxyl radical-induced DNA damage via antioxidant mechanism: an in vitro study
Source: Bot Stud. 2014 Feb 2;55:16. doi: 10.1186/1999-3110-55-16 (PMC5430338; doi:10.1186/1999-3110-55-16)

**Additional 5-** **The proposed reaction for aloe-emodin to scavenge** •**OH via SEPT**


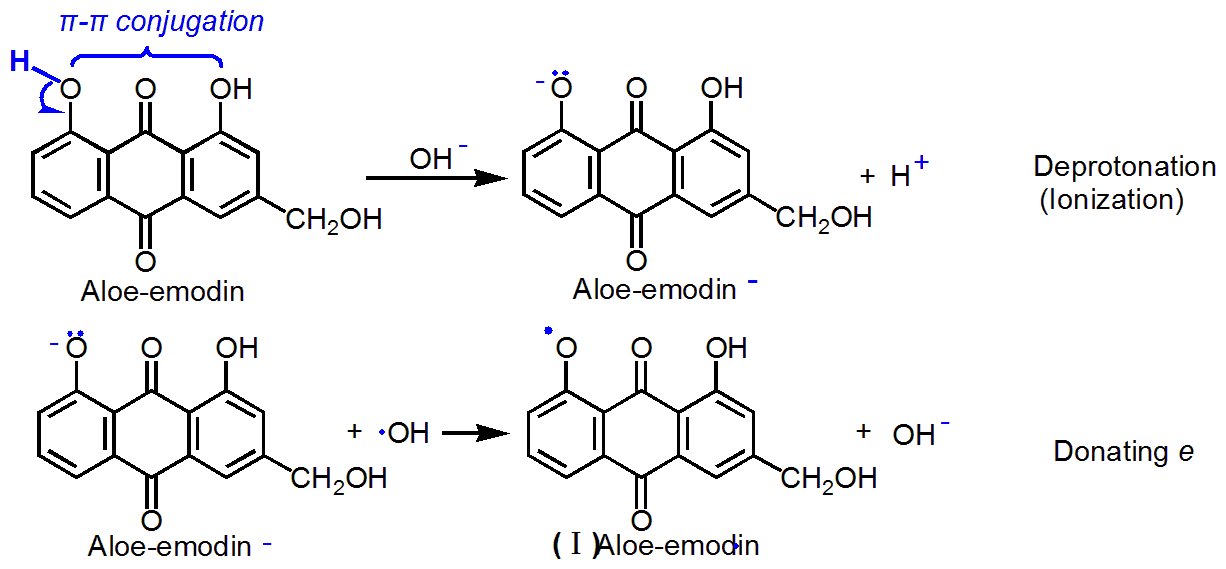

Supplement: Supplementary file 5 — Additional file 5:The proposed reaction for aloe-emodin to scavenge •OH via SEPT.(DOC 45 KB) [file 40529_2013_68_MOESM5_ESM.doc]

**Additional 6-The proposed reaction for aloe-emodin to scavenge** •**OH via HAT**

**
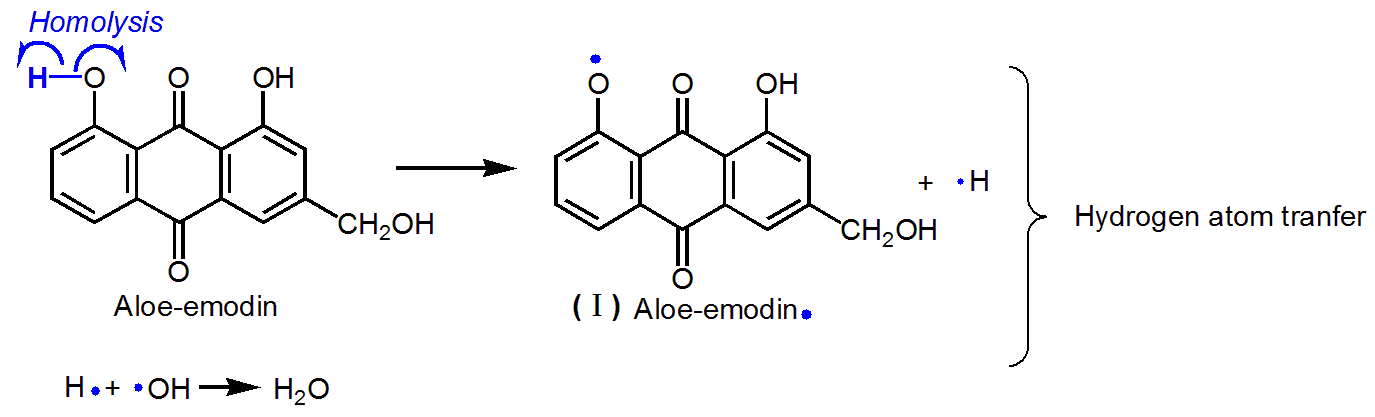
**

Supplement: Supplementary file 6 — Additional file 6:The proposed reaction for aloe-emodin to scavenge •OH via HAT.(DOC 40 KB) [file 40529_2013_68_MOESM6_ESM.doc]

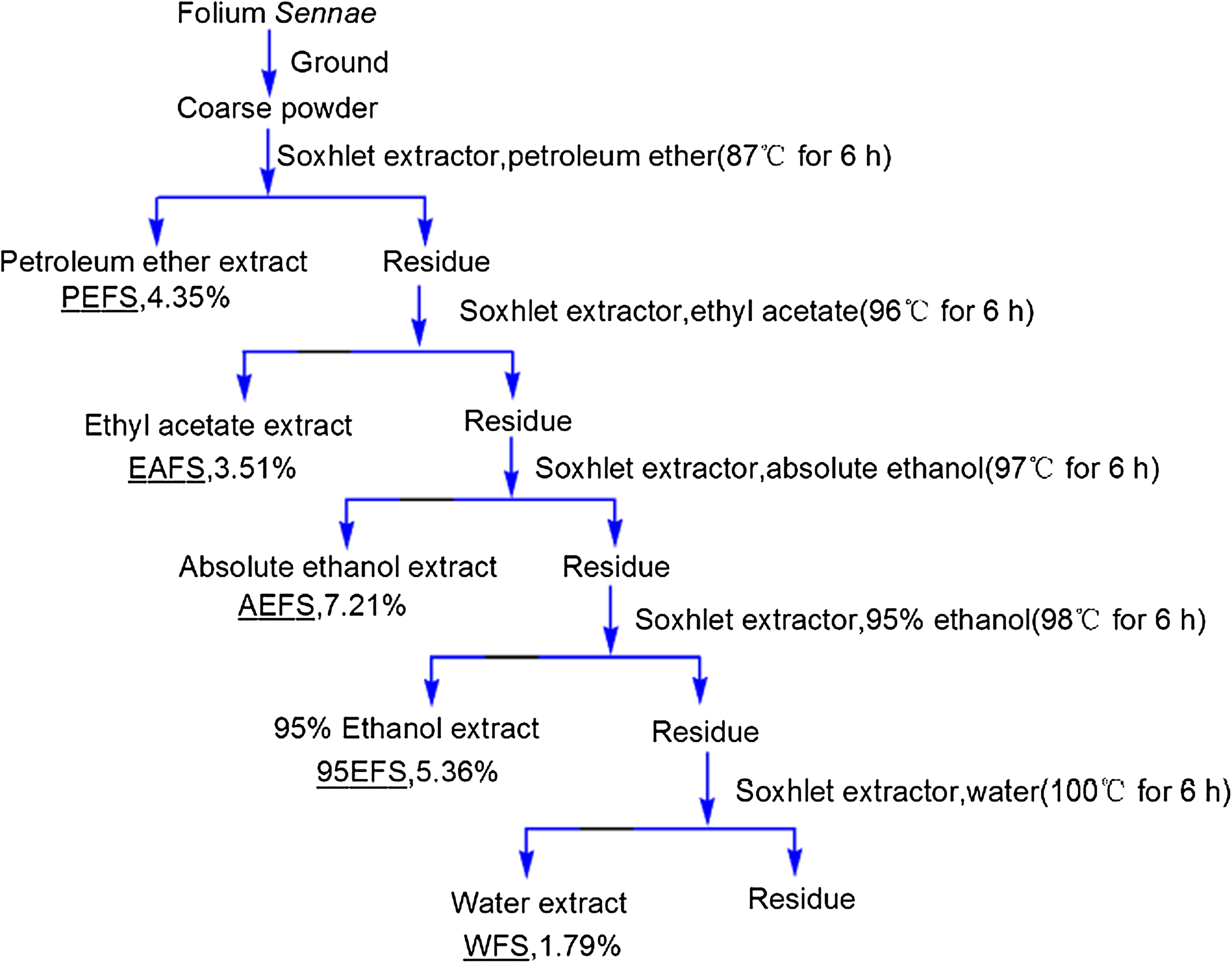

Supplement: Supplementary file 8 — Authors’ original file for figure 1 [file 40529_2013_68_MOESM8_ESM.tif]

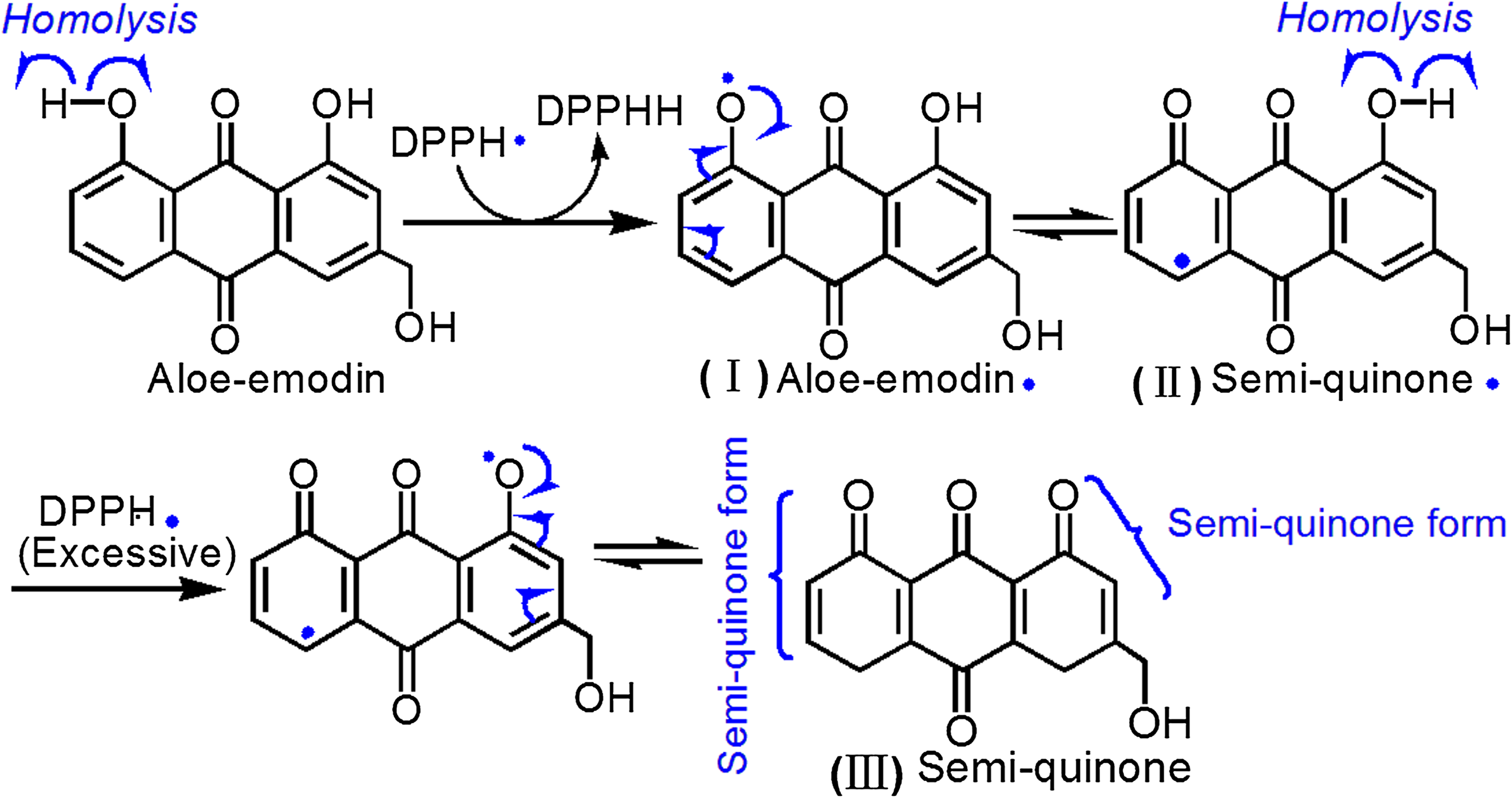

Supplement: Supplementary file 9 — Authors’ original file for figure 2 [file 40529_2013_68_MOESM9_ESM.tif]

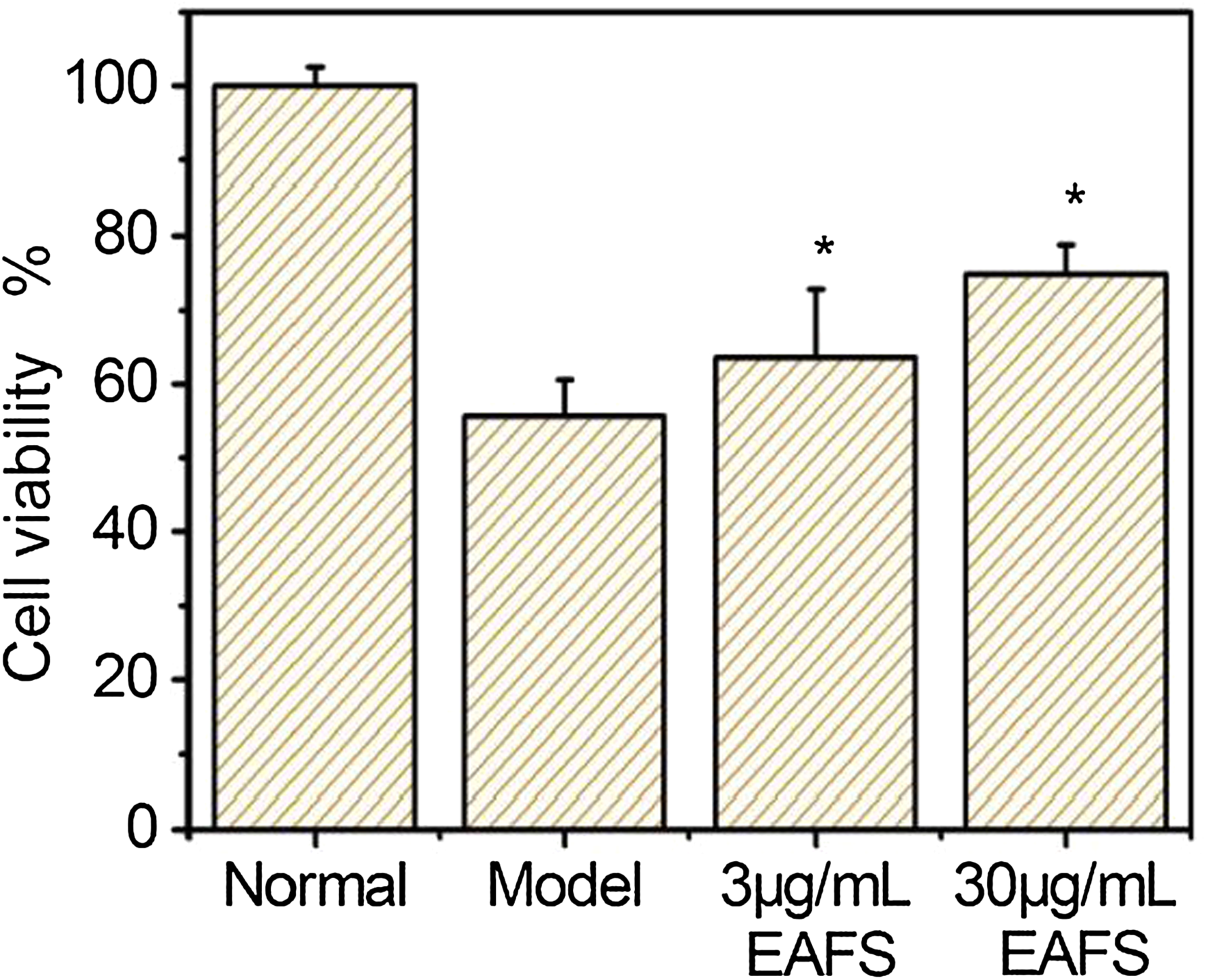

Supplement: Supplementary file 10 — Authors’ original file for figure 3 [file 40529_2013_68_MOESM10_ESM.tif]

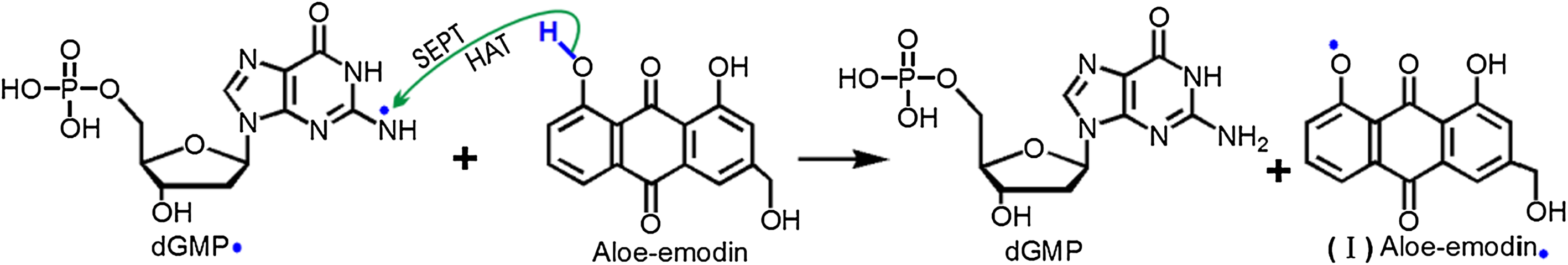

Supplement: Supplementary file 11 — Authors’ original file for figure 4 [file 40529_2013_68_MOESM11_ESM.tif]
