# Supplementary material for: Folium Sennae protects against hydroxyl radical-induced DNA damage via antioxidant mechanism: an in vitro study
Source: Bot Stud. 2014 Feb 2;55:16. doi: 10.1186/1999-3110-55-16 (PMC5430338; doi:10.1186/1999-3110-55-16)
Supplement: Supplementary file 7 — Additional file 7:The proposed reaction of •OH radical attack dGMP to form dGMP•.(DOC 42 KB) [file 40529_2013_68_MOESM7_ESM.doc]

**Additional 7-The proposed reaction of** •**OH radical attack dGMP to form dGMP**•

**
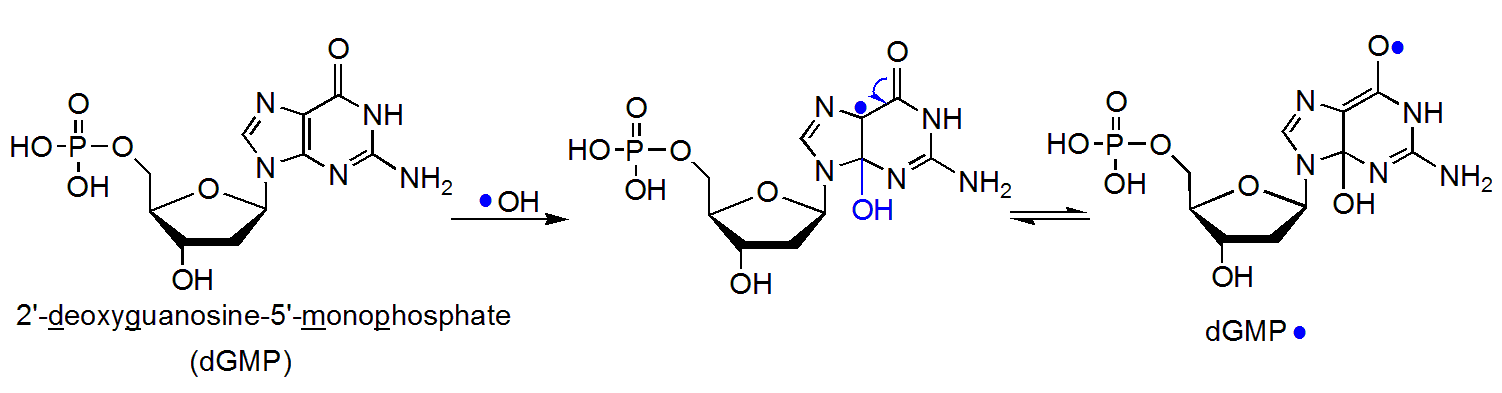
**

**References**

A6.1 Zheng RL, Wang CM, Lin CJ, Shi YM, Li J, Zhao CY et al. The earliest stage of carcinogenesis blocked by the fast repair of DNA transient damage. Acta Biophys Sinica 2012;**28**:185-199.
